# Supplementary material for: Gender-based homophily in collaborations across a heterogeneous scholarly landscape
Source: PLoS One. 2023 Apr 5;18(4):e0283106. doi: 10.1371/journal.pone.0283106 (PMC10075399; doi:10.1371/journal.pone.0283106)
Supplement: S1 File — The α values and p-values for each field and all the other descriptive statistics reported in this document are openly available on our project website http://eigenfactor.org/projects/gender_homophily. Because the raw publication data are provided by JSTOR under license to the authors, requests for the raw data should be made to JSTOR directly. Code for the analysis and plots is available at https://github.com/ysamwang/genderHomophily. The supplement includes additional details on the calculations for Fig 1, the JSTOR data set, the data cleaning procedure, and the Metropolis-Hastings sampler for the null distribution. We also provide additional tables for the main analysis, secondary analysis, and sensitivity analysis, as well as additional simulation studies. (PDF) [file pone.0283106.s001.pdf]

# Supplement: Gender-based homophily in collaborations across a heterogeneous scholarly landscape

Y. Samuel Wang, Carole J. Lee, Jevin D. West, Carl T. Bergstrom, Elena A. Erosheva

December 19, 2022

## 1 Interactive browser

An interactive browser of the Eigenfactor clustering and results for all top-level, composite, and terminal fields is available online: `http://eigenfactor.org/projects/gender_homophily`.

The browser provides a multiscale view of gender homophily across scholarly publishing. The size of the box indicates the size of a field relative to the top-level field. The grey boxes indicate nonexistent fields. Some fields such as ecology and evolution have many layers with many subfields. Smaller fields such as History have less subfields. The green colors indicates the Benjamini-Yekutieli adjusted p-values for comparing the expected alpha values with the observed alpha values. The observed alpha values are represented with a red line in the histogram graphs. The darker green colors are statistically significant. Lighter green is less so. For example, "Macroeconomics" has an expected alpha value around 0.03 and an observed alpha value of 0.07. The p-value indicated for this field is 0.030. The legend below provides the cutoff values for each color.

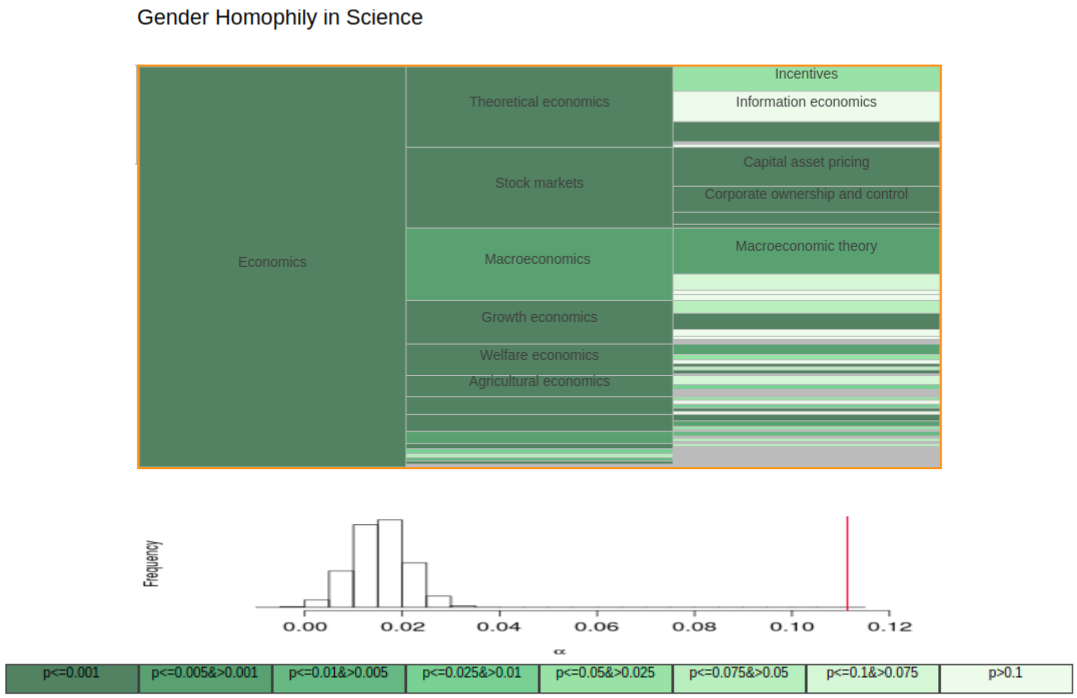


Figure 1: Example of the interactive browser for Economics and legend for how p-values for each field correspond to a color.

# 2 Measure of Homophily

Recall that $\alpha = p - q$ where $p$ is the probability that a randomly selected co-author of a randomly selected man authorship is a man and $q$ is the probability that a randomly selected co-author of a randomly selected woman authorship is a man.

We first give detailed calculations for the different values of $\alpha$ described in Section 2.1. Recall that we consider a corpus where all documents have only 2 authors. Let $n$ denote the total number of authorships and let $n_w$ and $n_m$ denote the number of woman and man authorships respectively. Note that there are $n/2$ total documents. To simplify the calculation, we let $\pi = n_w/n$ and assume that $\pi \leq 1/2$.

We first consider the case when the proportion of woman-man papers is $2\pi(1-\pi)$. Then, the number of men who co-authored with a women is $2\pi(1-\pi) \times n/2 = (n_w/n)(n_m/n)n = n_w n_m/n$, thus the number of men who co-author with other men is $n_m - n_w n_m/n = n_m(1 - n_w/n)$. Similarly, then number of women who co-authored with a man is $n_w n_m/n$ and the number of women who co-authored with a woman is $n_w(1 - n_m/n$. We then have that

$$p = \frac{n_m(1 - n_w/n)}{n_m} \qquad \text{and} \qquad q = \frac{n_w n_m/n}{n_w}. \tag{1}$$

Thus,

$$\begin{aligned} \alpha &= 1 - \frac{n_w}{n} - \frac{n_m}{n} = \frac{n - n_w - n_m}{n} \\ &= 0. \end{aligned} \tag{2}$$

Now consider the case where the number of woman-man papers is 0 so that men only co-author with men and women only co-author with women. Then,

$$p = 1 \qquad \text{and} \qquad q = 0. \tag{3}$$

and $\alpha = 1$. Finally consider the case where we have the maximum number of woman-man papers, $2\pi$. Then, the number of men who co-author with women is $2\pi \times n/2 = n_w$ and the number of men who co-author with men is $n_m - n_w$. Since we assume that $n_w \leq n_m$, the number of women who co-author with men is 0. We then have:

$$p = \frac{n_m - n_w}{n_m} \qquad \text{and} \qquad q = 1. \tag{4}$$

Thus,

$$\alpha = 1 - \frac{n_w}{n_m} - 1 = -\frac{n_w/n}{n_m/n} \quad = -\frac{\pi}{1-\pi}. \tag{5}$$

Figure 2: Two fields with four papers each. Each square represents a paper, each circle represents an authorship, and color indicates gender (blue is man, red is woman).

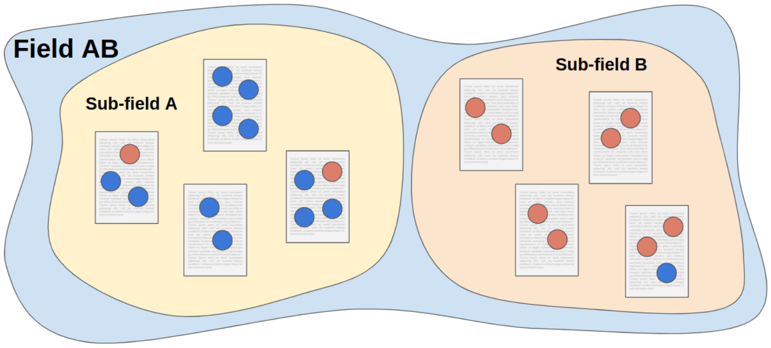

We now calculate the $\alpha$ for the example given in Figure 1 in the main text. For Field A, there are 11 men and 2 women. To calculate $p$ in the following equation, we calculate the proportion of man co-authors for each man authorship and then take the average. The values in the equation correspond to authorships from left to right. To calculate $q$, we calculate the proportion of man co-authors for each woman authorship and then take the average–again from left to right.

$$\begin{aligned} p &= \frac{1}{11}\left[\left(\frac{1}{2}+\frac{1}{2}\right)+(1+1)+(1+1+1+1)+\left(\frac{2}{3}+\frac{2}{3}+\frac{2}{3}\right)\right] = 9/11 \\ q &= \frac{1}{2}\left[(1)+(1)\right] = 1 \\ \alpha &= -2/11 \end{aligned} \tag{6}$$

For Field B, there is 1 man and 8 women.

$$\begin{aligned} p &= \frac{1}{1}\left[0\right] = 0 \\ q &= \frac{1}{8}\left[(0+0)+(0+0)+(0+0)+\left(\frac{1}{2}+\frac{1}{2}\right)\right] = 1/8 \\ \alpha &= -1/8 \end{aligned} \tag{7}$$

For the Fields $A$ and $B$ combined, there are 12 men and 10 women.

$$\begin{aligned} p &= \frac{1}{12}\left[\left(\frac{1}{2}+\frac{1}{2}\right)+(1+1)+(1+1+1+1)+\left(\frac{2}{3}+\frac{2}{3}+\frac{2}{3}\right)+(0)\right] = 3/4 \\ q &= \frac{1}{10}\left[(1)+(1)+(0+0)+(0+0)+(0+0)+\left(\frac{1}{2}+\frac{1}{2}\right)\right] = 3/10 \\ \alpha &= 9/20 \end{aligned} \tag{8}$$

Finally, in Section 2.1 of the main manuscript, we describe a concrete interpretation of $\alpha$ displayed in the "WM-Papers" column of Table 1. In particular, we assume a field consists of 100 2-author papers and let $\pi$ and $1-\pi$ be the proportion of woman and man authorships respectively. We can calculate the number (which may be fractional) of woman-man papers ($WM$), man-man papers ($MM$) and woman-woman papers ($WW$) which would result in a specific $\alpha$. In this setting $p = 2 \times MM/(200(1-\pi))$ and $q = WM/(200\pi)$. Thus,

$$\alpha = \frac{2MM}{200(1-\pi)} - \frac{WM}{200\pi}.$$

Note that $MM + WM + WW = 100$ because there are 100 papers total, and $2WW + MW = 200\pi$ since $\pi$ is the proportion of woman authorships. Thus, solving for $WM$, $MM$, and $WW$ then yields:

$$\begin{aligned} WM &= 200 \times (1-\alpha)\,\pi(1-\pi) \\ MM &= 100(1-\pi)\left[1-(1-\alpha)\pi\right] \\ WW &= 100\pi\left[1-(1-\alpha)(1-\pi)\right]. \end{aligned}$$

# 3 JSTOR Description

Table 1 shows the size of each of the 24 top level fields identified by the map equation. The values are calculated for all papers published in or after 1960. Note that the table describes the data prior to the data cleaning procedure, so counts of authorships, papers, terminal fields and composite fields shown here may differ from those given in the main manuscript which refer to data after the cleaning procedure. Specifically, Classics, Law, and Philosophy have entire terminal fields which are removed by the cleaning procedure. Table 2 presents the structural characteristics of each top level field. For the multi-author columns, we report the proportion amongst all authorships on a multi-author paper; e.g., all woman authorships on multi-authored papers divided by the total count of authorships on multi-authored papers. For the intraclass correlation (ICC) of individuals with unimputed genders, we use the $\rho_{AOV}$ statistic from (Ridout et al., 1999). This gives a measure of how unimputed authorships cluster by paper. Anecdotally, unimputed authorships are often names which have been Romanized. Thus a high ICC may indicate homophily by race or ethnicity.

Table 1: Size of each of the top level fields identified by the map equation hierarchical clustering

| Label | Authors (Count) | Papers (Count) | Terminal Fields | Composite Fields |
|---|---|---|---|---|
| Anthropology | 37588 | 30499 | 63 | 8 |
| Classical studies | 10596 | 9061 | 37 | 8 |
| Cognitive science | 15715 | 5553 | 14 | 3 |
| Demography | 9653 | 5509 | 20 | 2 |
| Ecology and evolution | 264853 | 116327 | 257 | 56 |
| Economics | 95934 | 59096 | 136 | 28 |
| Education | 40188 | 23065 | 42 | 10 |
| History | 26449 | 24043 | 49 | 6 |
| Law | 23974 | 19779 | 105 | 16 |
| Mathematics | 18348 | 14125 | 46 | 9 |
| Molecular & Cell biology | 382971 | 92528 | 178 | 44 |
| Mycology | 7469 | 3679 | 16 | 1 |
| Operations research | 13716 | 7780 | 18 | 4 |
| Organizational and marketing | 34254 | 17963 | 68 | 4 |
| Philosophy | 21738 | 19126 | 46 | 8 |
| Physical anthropology | 29693 | 16703 | 32 | 10 |
| Plant physiology | 9159 | 5436 | 21 | 3 |
| Political science - international | 15283 | 11835 | 34 | 2 |
| Political science-US domestic | 12581 | 7824 | 37 | 6 |
| Pollution and occupational health | 50967 | 12359 | 24 | 1 |
| Probability and Statistics | 37471 | 22094 | 90 | 23 |
| Radiation damage | 14118 | 4215 | 14 | 5 |
| Sociology | 57146 | 31662 | 94 | 21 |
| Veterinary medicine | 17756 | 4796 | 19 | 2 |

Table 2: The structural characteristics of each top level field identified by the map equation hierarchical clustering. The "Prop Single-Author" columns display the proportion of papers (authorships) which are single-authored out of all papers (authorships). The "Single-author" ("Multi-Author") column give the proportions of all authorships on single-authored (multi-authored) papers which were imputed a gender based on first name. W- Woman; M- Man; U- Unimputed. The "ICC" column displays the intraclass correlation of unimputed authorships on multi-author papers.

| | Prop Single Author | | Single-Author | | | Multi-Author | | | |
|---|---|---|---|---|---|---|---|---|---|
| Label | Papers | Auth | % W | % M | % U | % W | % M | % U | ICC |
| Anthropology | 0.86 | 0.70 | 0.27 | 0.63 | 0.10 | 0.28 | 0.61 | 0.12 | 0.10 |
| Classical studies | 0.93 | 0.79 | 0.22 | 0.70 | 0.08 | 0.27 | 0.65 | 0.08 | 0.05 |
| Cognitive science | 0.25 | 0.09 | 0.29 | 0.64 | 0.07 | 0.28 | 0.62 | 0.10 | 0.09 |
| Demography | 0.57 | 0.32 | 0.24 | 0.61 | 0.15 | 0.30 | 0.53 | 0.16 | 0.11 |
| Ecology and evolution | 0.37 | 0.16 | 0.14 | 0.79 | 0.08 | 0.20 | 0.70 | 0.11 | 0.10 |
| Economics | 0.55 | 0.34 | 0.08 | 0.81 | 0.11 | 0.11 | 0.77 | 0.12 | 0.10 |
| Education | 0.55 | 0.31 | 0.35 | 0.58 | 0.07 | 0.41 | 0.50 | 0.08 | 0.07 |
| History | 0.92 | 0.84 | 0.24 | 0.70 | 0.06 | 0.23 | 0.69 | 0.08 | 0.05 |
| Law | 0.85 | 0.70 | 0.17 | 0.78 | 0.06 | 0.22 | 0.71 | 0.06 | 0.05 |
| Mathematics | 0.75 | 0.58 | 0.06 | 0.76 | 0.18 | 0.06 | 0.73 | 0.21 | 0.19 |
| Molecular & Cell biology | 0.14 | 0.03 | 0.19 | 0.70 | 0.10 | 0.23 | 0.61 | 0.16 | 0.09 |
| Mycology | 0.45 | 0.22 | 0.20 | 0.71 | 0.09 | 0.22 | 0.65 | 0.13 | 0.08 |
| Operations research | 0.48 | 0.27 | 0.05 | 0.81 | 0.14 | 0.08 | 0.72 | 0.20 | 0.21 |
| Organizational and marketing | 0.40 | 0.21 | 0.18 | 0.72 | 0.10 | 0.19 | 0.68 | 0.12 | 0.12 |
| Philosophy | 0.89 | 0.78 | 0.09 | 0.82 | 0.08 | 0.10 | 0.78 | 0.11 | 0.12 |
| Physical anthropology | 0.66 | 0.37 | 0.22 | 0.72 | 0.06 | 0.22 | 0.68 | 0.09 | 0.07 |
| Plant physiology | 0.53 | 0.31 | 0.13 | 0.79 | 0.08 | 0.17 | 0.72 | 0.10 | 0.07 |
| Political science - international | 0.78 | 0.60 | 0.16 | 0.74 | 0.10 | 0.17 | 0.73 | 0.10 | 0.08 |
| Political science-US domestic | 0.57 | 0.35 | 0.17 | 0.76 | 0.07 | 0.18 | 0.75 | 0.07 | 0.05 |
| Pollution and occupational health | 0.22 | 0.05 | 0.24 | 0.65 | 0.11 | 0.31 | 0.53 | 0.17 | 0.19 |
| Probability and Statistics | 0.54 | 0.32 | 0.08 | 0.75 | 0.17 | 0.13 | 0.69 | 0.19 | 0.20 |
| Radiation damage | 0.23 | 0.07 | 0.22 | 0.66 | 0.11 | 0.22 | 0.62 | 0.16 | 0.14 |
| Sociology | 0.52 | 0.29 | 0.30 | 0.63 | 0.08 | 0.38 | 0.53 | 0.08 | 0.07 |
| Veterinary medicine | 0.26 | 0.07 | 0.27 | 0.60 | 0.12 | 0.25 | 0.59 | 0.15 | 0.22 |

The plots below show how the following quantities have changed over time for each top level fields- average number of authors per paper, proportion of papers with multiple authors, and the imputed gender proportions. The values are calculated on the data before the data-cleaning procedure which removes authorship instances with unimputed genders.

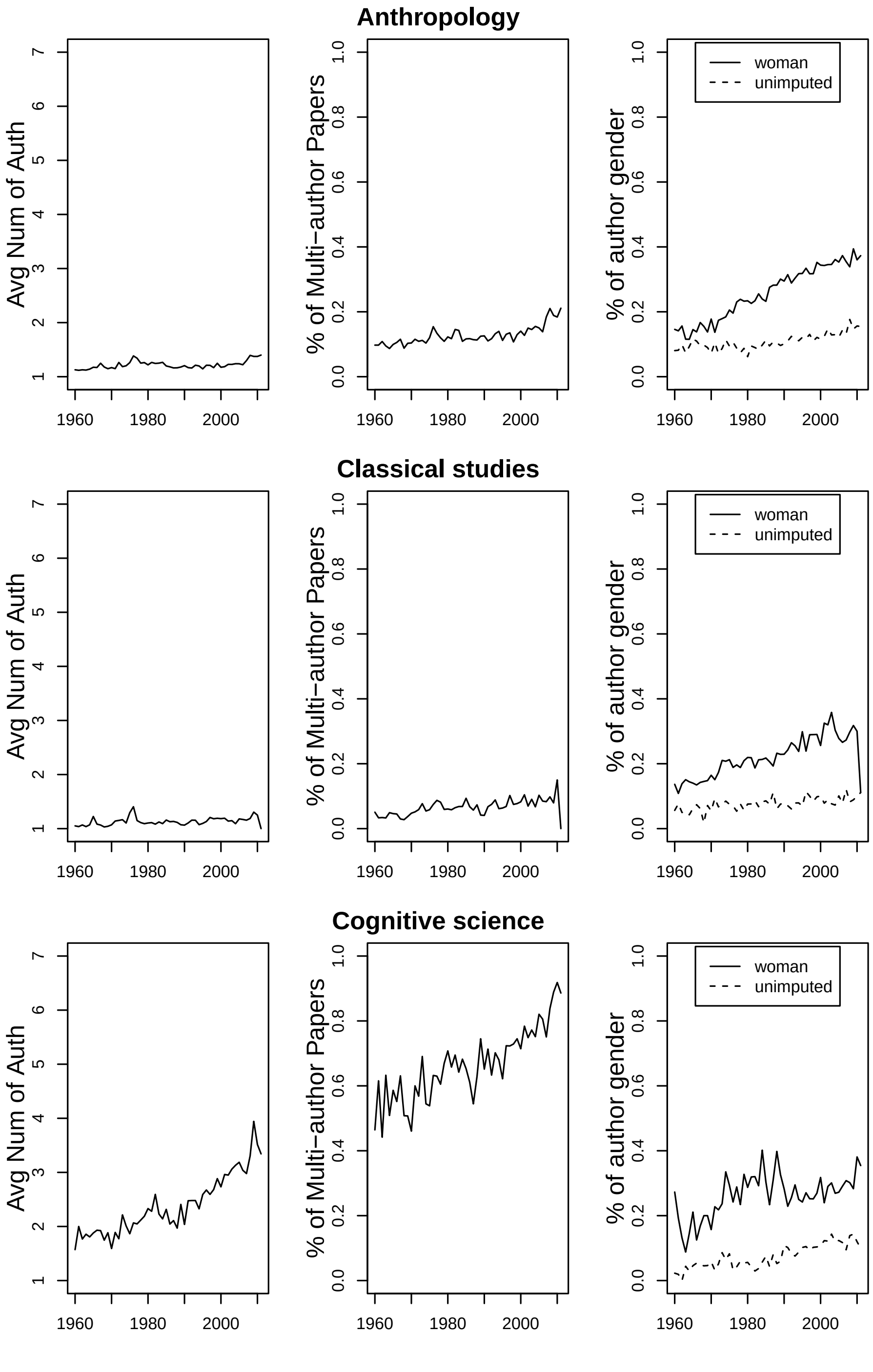
Anthropology
Classical studies
Cognitive science
Avg Num of Auth
% of Multi−author Papers
% of author gender
woman
unimputed
1960
1980
2000
1
2
3
4
5
6
7
0.0
0.2
0.4
0.6
0.8
1.0

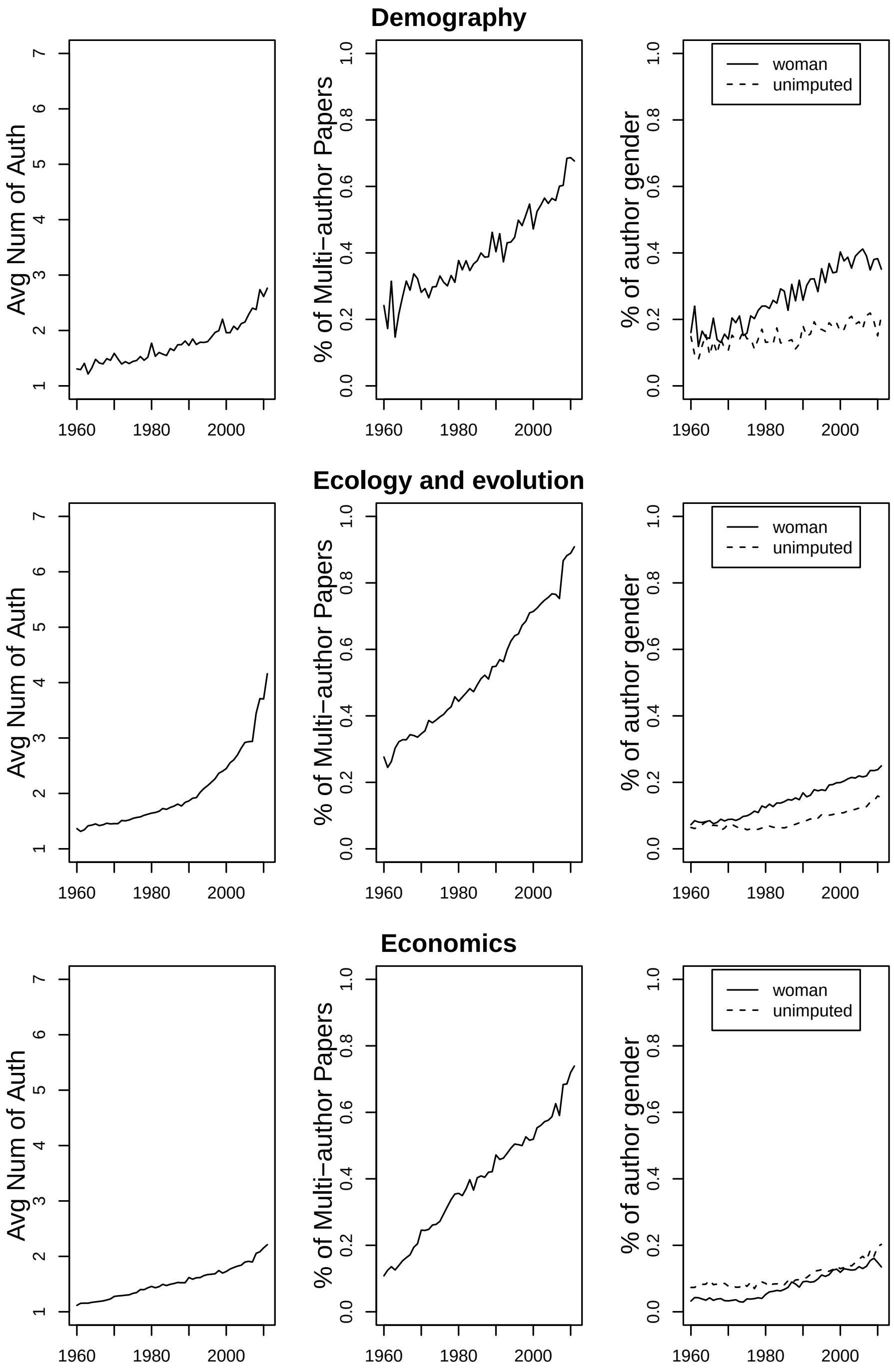
Demography
Ecology and evolution
Economics
Avg Num of Auth
% of Multi−author Papers
% of author gender
woman
unimputed
1960
1980
2000

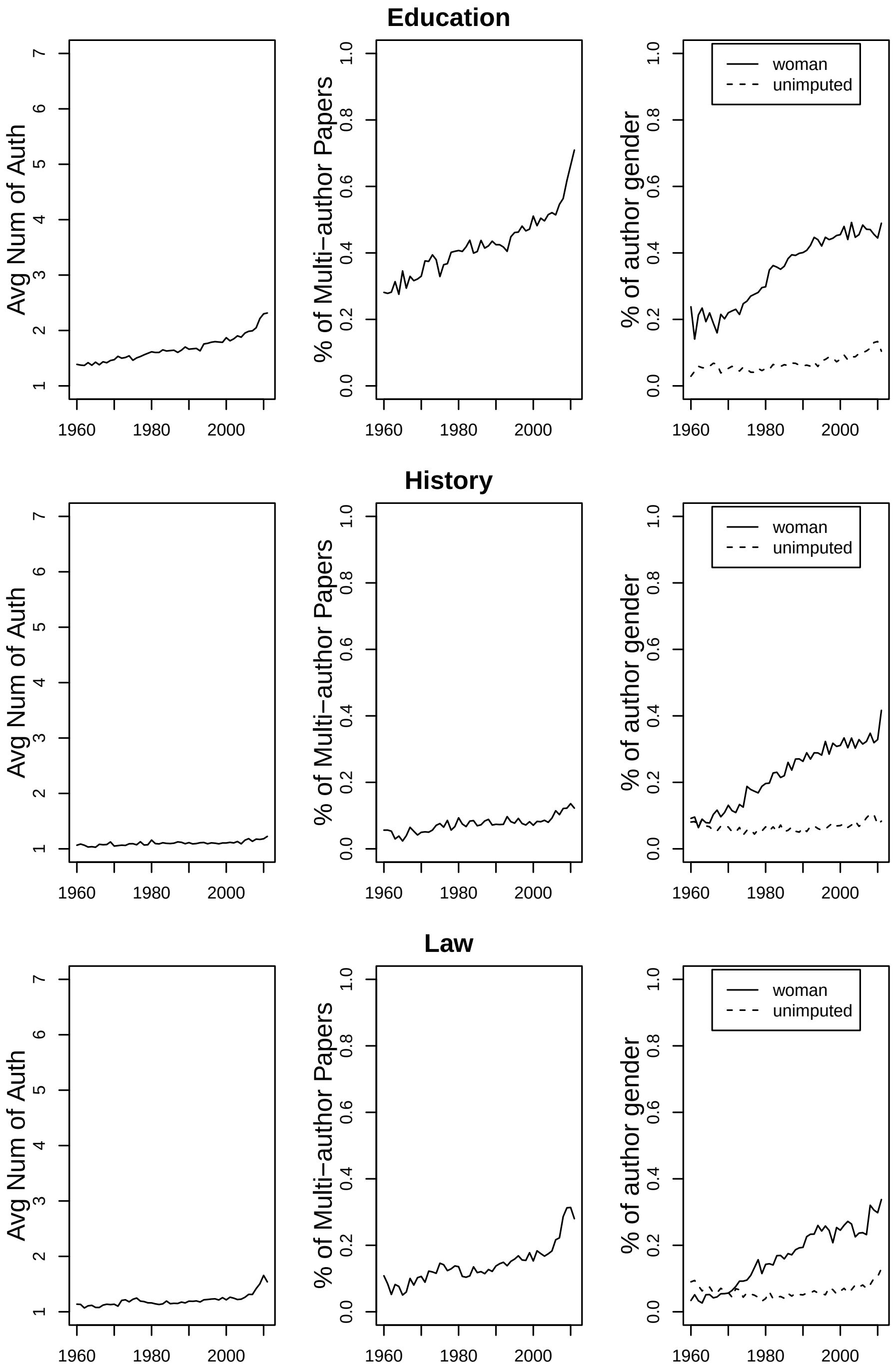
Education
History
Law
Avg Num of Auth
% of Multi−author Papers
% of author gender
woman
unimputed
1960
1980
2000

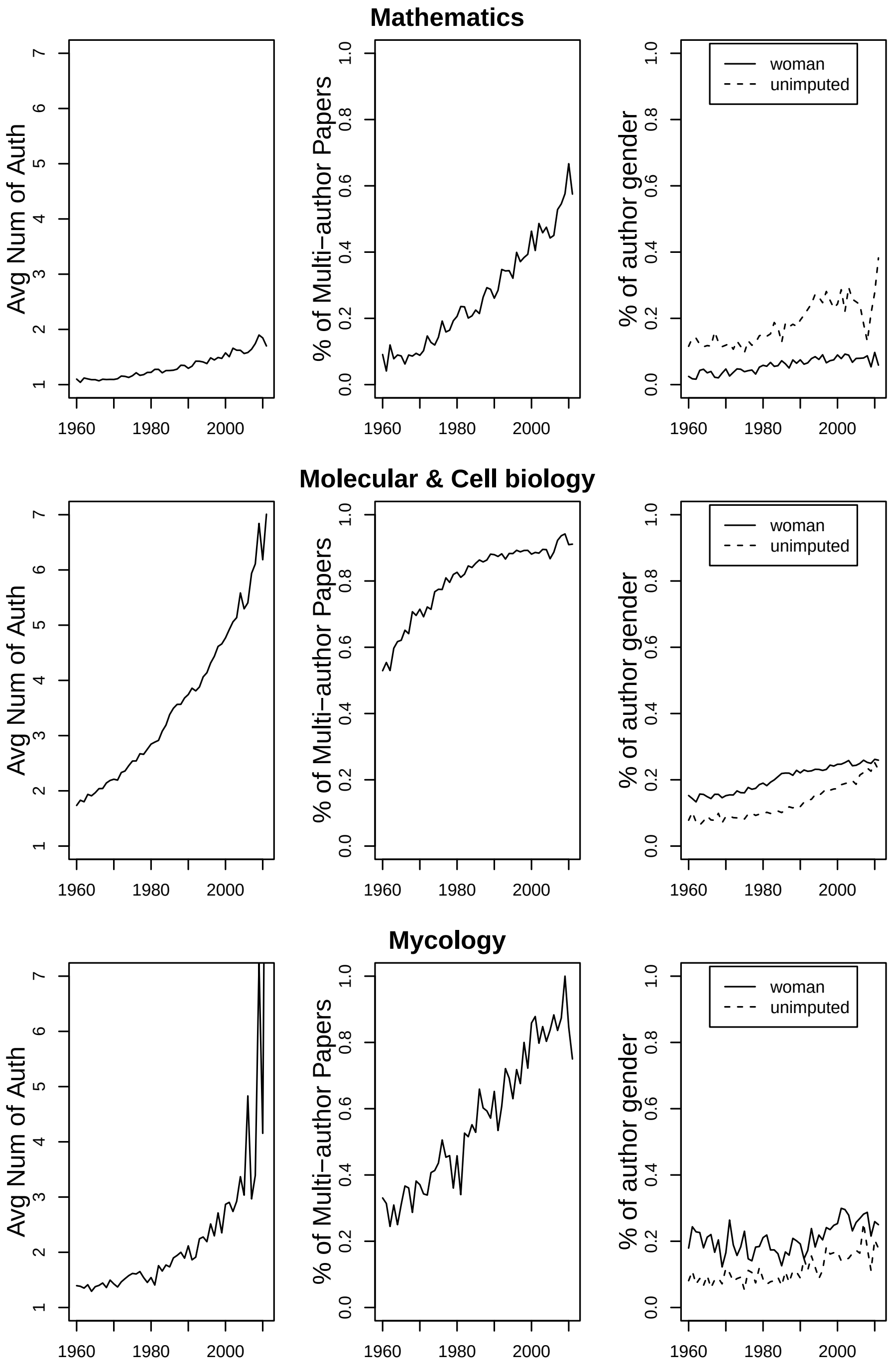
Mathematics
Molecular & Cell biology
Mycology
Avg Num of Auth
% of Multi−author Papers
% of author gender
woman
unimputed
1960
1980
2000

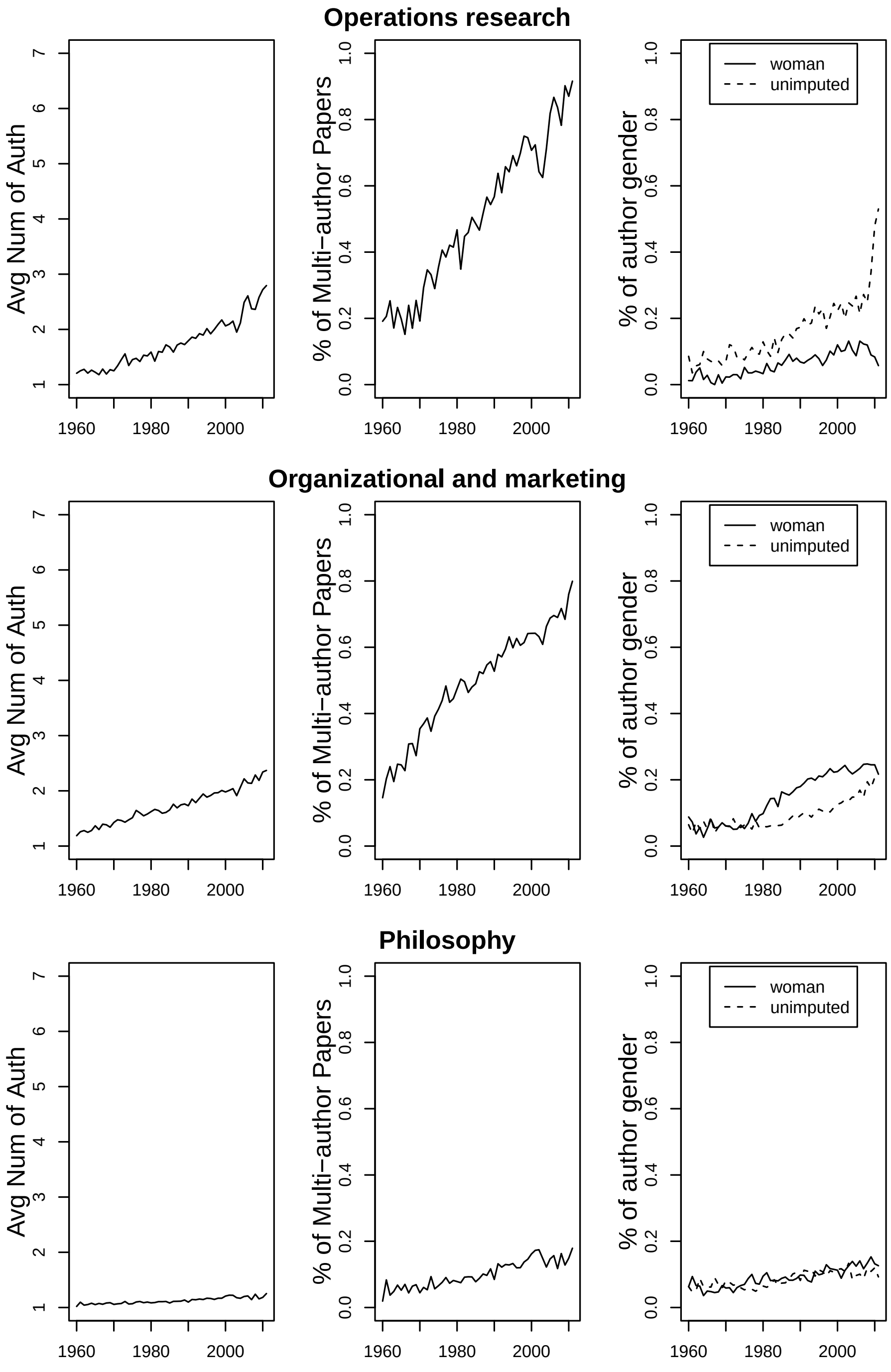
Operations research
Organizational and marketing
Philosophy
Avg Num of Auth
% of Multi−author Papers
% of author gender
woman
unimputed
1960
1980
2000

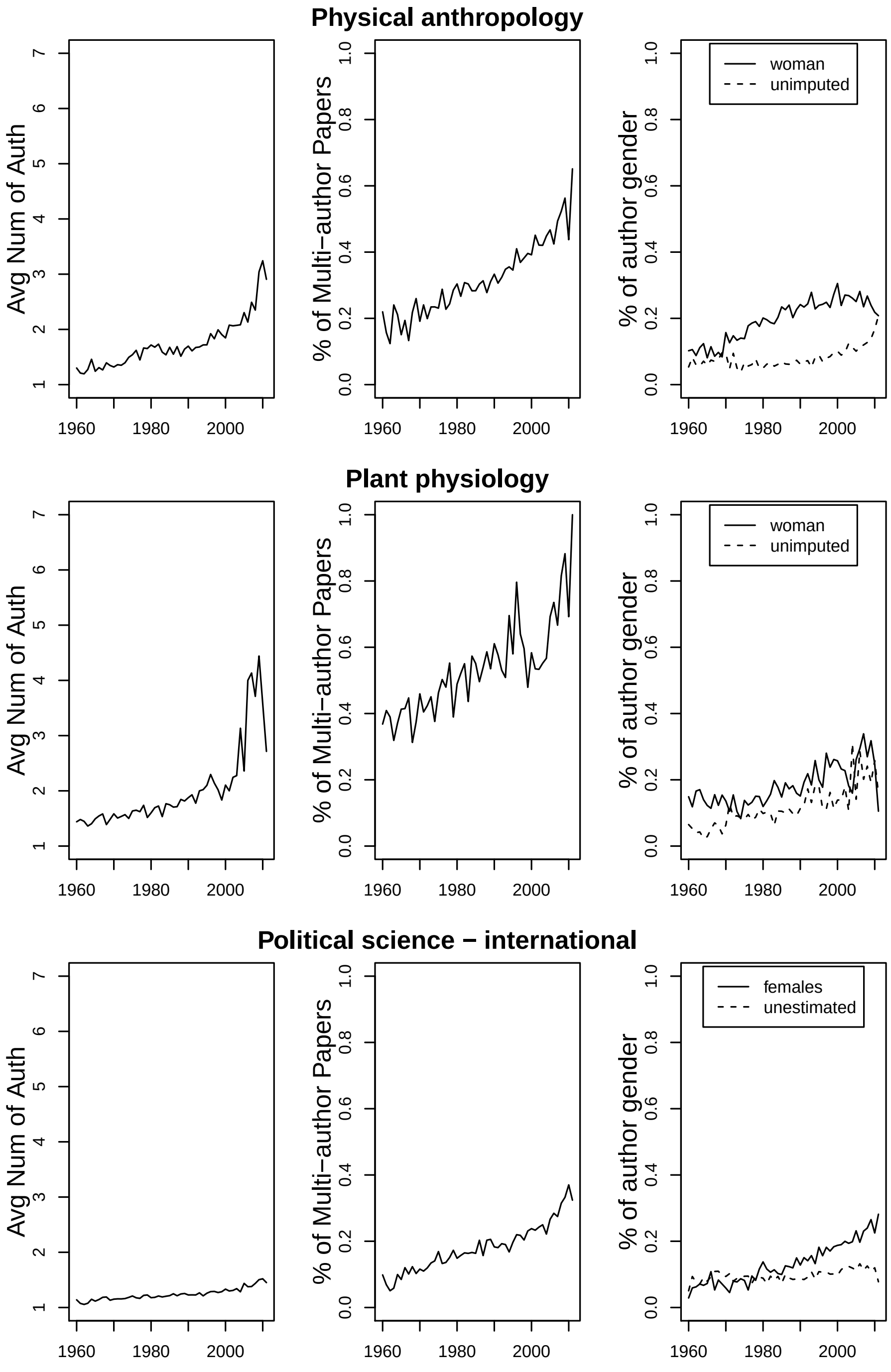
Physical anthropology
Avg Num of Auth
% of Multi−author Papers
% of author gender
woman
unimputed
Plant physiology
Avg Num of Auth
% of Multi−author Papers
% of author gender
woman
unimputed
Political science − international
Avg Num of Auth
% of Multi−author Papers
% of author gender
females
unestimated
1960
1980
2000

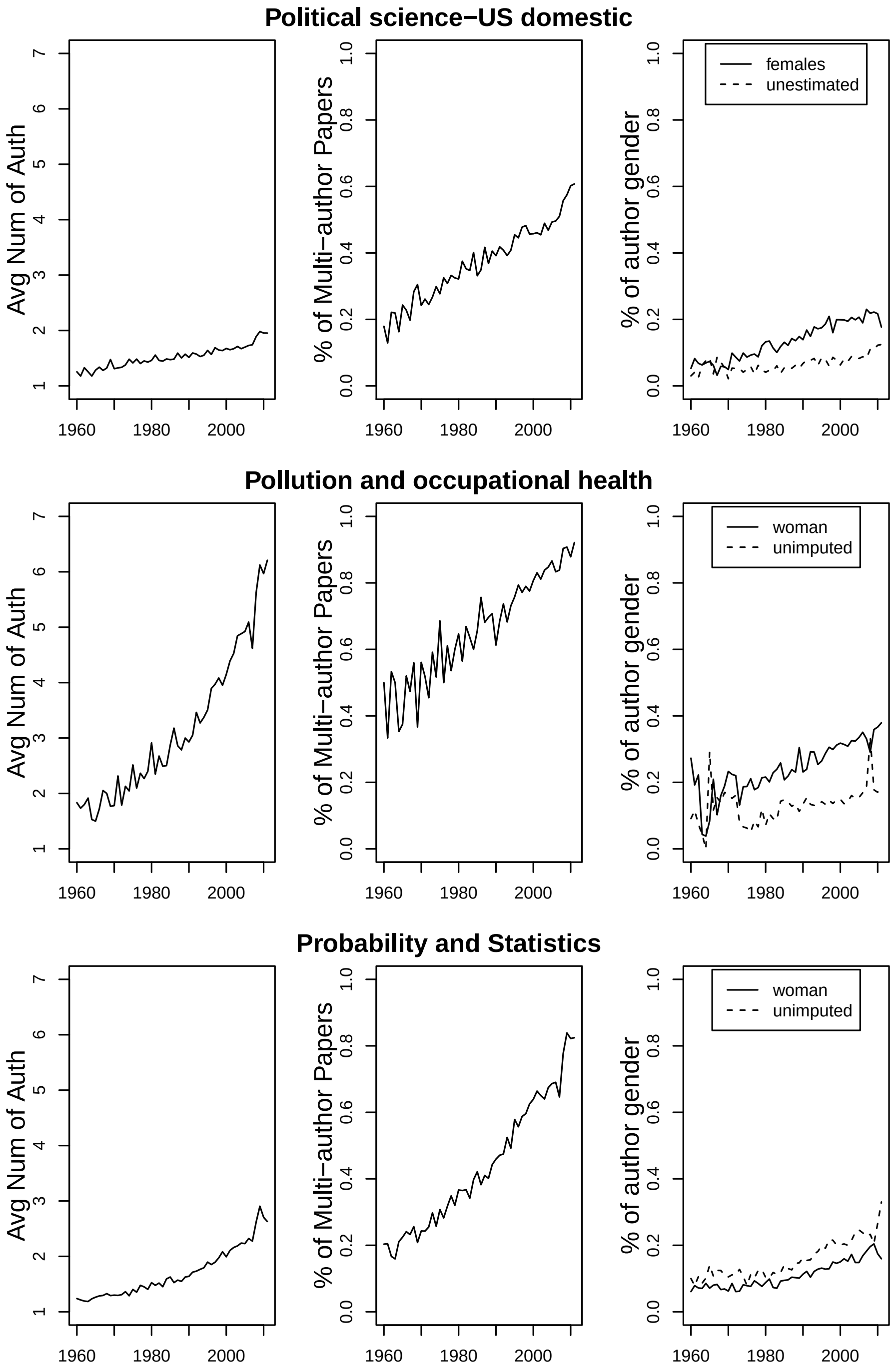
Political science−US domestic
Avg Num of Auth
% of Multi−author Papers
% of author gender
females
unestimated
Pollution and occupational health
Avg Num of Auth
% of Multi−author Papers
% of author gender
woman
unimputed
Probability and Statistics
Avg Num of Auth
% of Multi−author Papers
% of author gender
woman
unimputed
1960
1980
2000

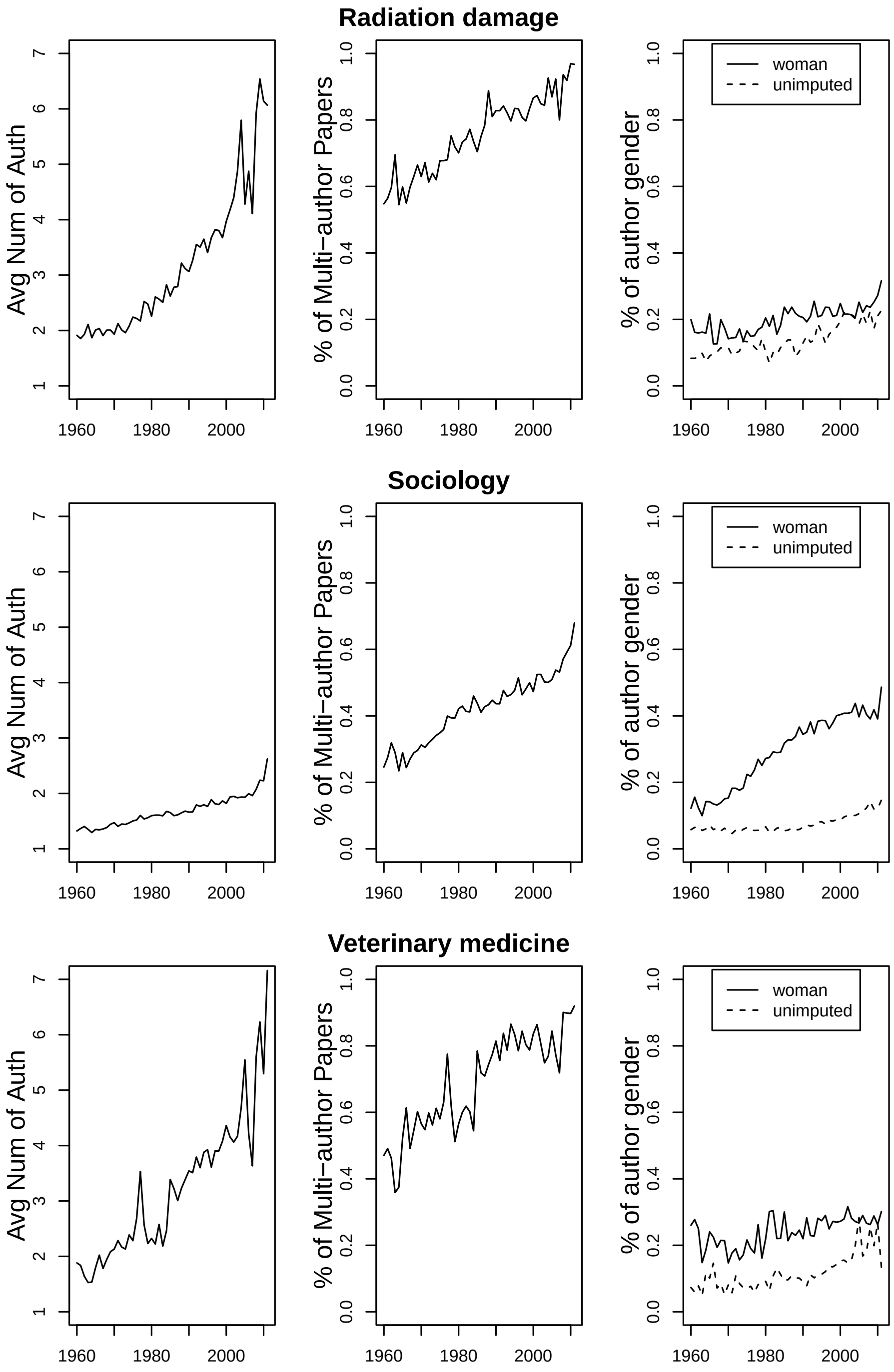
Radiation damage
Sociology
Veterinary medicine
Avg Num of Auth
% of Multi−author Papers
% of author gender
woman
unimputed
1960
1980
2000

# 4 Data Cleaning Procedures

For the main analysis, we impute gender indicators for authorships with first names that are used for a single gender with at least 95% frequency in either the U.S. Social Security records or in the `genderizeR` database. We consider the gender indicator to be missing for authorships that either do not appear in those databases or are not used with at least 95% frequency for one gender. We subsequently remove authorships with unimputed genders from our main analysis. For instance, a paper with 2 women, 1 man, and 2 unimputed authorships is treated as an article with 2 woman and 1 man authorships. This removal results in some articles which originally had multiple authors becoming single author papers, which are excluded from the analysis. The following table shows the proportion of authorships and papers which are lost *solely* due to unimputed genders. The denominator only includes papers which have multiple authors which were published from 1960-2011. The % unimputed column is the % of authors for which we do not impute a gender indicator. The % Lost column is the % of authors (or papers) which are lost after removing the authorships with unimputed gender indicators and then removing the resulting single author papers. For authorships, this percentage includes the authorships with unimputed genders.

Table 3: Data reduction due to unimputed gender indicators

| | Prop Authors with | Authors | | Papers | |
|---|---|---|---|---|---|
| Label | Unimputed Gender | Remaining | Prop Lost | Remaining | Prop Lost |
| Anthropology | 0.12 | 9326 | 0.17 | 3466 | 0.15 |
| Classical studies | 0.08 | 1976 | 0.11 | 610 | 0.09 |
| Cognitive science | 0.10 | 12510 | 0.13 | 3814 | 0.07 |
| Demography | 0.16 | 5069 | 0.22 | 1930 | 0.17 |
| Ecology and evolution | 0.11 | 192091 | 0.13 | 66152 | 0.08 |
| Economics | 0.12 | 51691 | 0.19 | 22178 | 0.15 |
| Education | 0.08 | 24356 | 0.12 | 9396 | 0.09 |
| History | 0.08 | 3699 | 0.12 | 1596 | 0.11 |
| Law | 0.06 | 6526 | 0.10 | 2765 | 0.09 |
| Mathematics | 0.21 | 5319 | 0.31 | 2459 | 0.25 |
| Molecular & Cell biology | 0.16 | 303761 | 0.18 | 73357 | 0.07 |
| Mycology | 0.13 | 4828 | 0.17 | 1759 | 0.11 |
| Operations research | 0.20 | 7217 | 0.28 | 3025 | 0.21 |
| Organizational and marketing | 0.12 | 22299 | 0.18 | 9137 | 0.13 |
| Philosophy | 0.11 | 3897 | 0.18 | 1770 | 0.15 |
| Physical anthropology | 0.09 | 16463 | 0.12 | 5175 | 0.07 |
| Plant physiology | 0.10 | 5388 | 0.14 | 2287 | 0.10 |
| Political science - international | 0.10 | 5128 | 0.16 | 2247 | 0.14 |
| Political science-US domestic | 0.07 | 7269 | 0.11 | 3068 | 0.09 |
| Pollution and occupational health | 0.17 | 39703 | 0.18 | 8845 | 0.06 |
| Probability and Statistics | 0.19 | 18763 | 0.27 | 7600 | 0.21 |
| Radiation damage | 0.16 | 10710 | 0.18 | 2902 | 0.09 |
| Sociology | 0.08 | 35858 | 0.12 | 13600 | 0.09 |
| Veterinary medicine | 0.15 | 13741 | 0.17 | 3275 | 0.06 |
| Total | 0.14 | 807588 | 0.16 | 252413 | 0.11 |

In our analysis, we do not address bias which may be induced if one gender is more likely to be unidentified than the other. Larivière Larivière et al. (2013) hand-check a sample of 1000 authorships randomly selected across all fields. For names for which no prior records existed, the proportions of men and women—.68 and .32 respectively (Larivière et al., 2013, Table S6)—were consistent with the proportions of men and women in the classified names–.69 and .31 of respectively (these are taken from (Larivière et al., 2013, Table S3) where we divide the proportion of identified man and woman author-name combinations by the total identified author-name combinations). In names which were not classified due to prevalent use for both genders, men were slightly overrepresented (.79 and .21).

# 5 Sampler Details

Recall from the main text, that for each authorship $a$ in the set of all authorships $A$, we let $f_a$ denote the terminal field, $d_a$ denote the document to which $a$ is assigned, and $y_a$ denotes the publication year of document $d_a$. We denote the entire configuration of all authorships as $X = \{f_a, d_a\}_{a\in A}$ and denote the configuration which we actually observe in the data as $X^*$. We use a Markov Chain Monte Carlo Metropolis-Hastings sampler to draw samples from the gender-blind null distribution:

$$P(X) = \frac{\mathbb{I}_{\{X\sim X^*\}} \prod_{a\in A} \left[p_{f_a, f_a^*} Z(y_a, y_a^\star)\right]}{\sum_{X'\sim X^*} \left(\prod_{a\in A} p_{f'_a, f_a^*} Z(y_a, y_a^\star)\right)}. \tag{9}$$

where the equivalence relationship $X \sim X^*$ indicates that the number of total authorships per terminal field, the total numbers of man and woman authorships, and the number of authorships per paper is the same in $X$ and $X^*$. Also, recall that:

$$Z(y_a, y_a^\star) = \begin{cases} 1 & \text{if } |y_a - y_a^\star| \leq 1 \\ (\frac{3}{4})^{|y_a - y_a^\star|^2} & \text{if } |y_a - y_a^\star| > 1 \end{cases}, \tag{10}$$

so that configurations where an authorship is swapped to a document with a similar publication year are much more likely than configurations where an authorship is swapped to a document with a very different publication year.

Define a permutation cycle of length $l$ to be a set of authorships $\{a_1, a_2, \dots a_l\}$ in which $a_i$ is reassigned to the current terminal field and document of $a_{i+1}$ and $a_l$ is reassigned the current terminal field and document of $a_1$. Any hypothetical configuration of authorship assignments $X$ can be decomposed into disjoint permutation cycles of the observed data $X^*$. The sampling procedure starts with the observed assignments of authorships to papers within terminal fields and generates assignments $X^{(t)}$, $t = 1, \dots, T$ by successively modifying the current state by a series of permutation cycles. We generate a proposal for each of these cycles by first randomly selecting a cycle length $l$ from a geometric distribution. Then, $l$ specific authorships are selected to form the permutation cycle. This proposed permutation cycle is then accepted or rejected with the appropriate Metropolis-Hastings probability.

For $i \in F$, let $\Lambda(i) = \{r \in A : p_{f_r i} > 0\}$, the authorships in a terminal field where any authorship originally from terminal field $i$ could be re-assigned.

The length of the proposed cycle $l \sim$ geometric($\pi$), where $\pi$ is a tuning parameter which regulates the average cycle length. A larger value of $\pi$ will yield longer cycles resulting in larger changes in the proposal but a lower probability of acceptance; a smaller value of $\pi$ will yield shorter cycles resulting in smaller changes in the proposal but a higher probability of acceptance. In general, the maximum length of a permutation cycle in the decomposition could be up to $|A|$, the number of authorships in our corpus. Thus, any distribution which has positive support over $1 \dots |A|$ would be sufficient for irreducibility. Under this scheme proposed in Algorithm 1, $P(X^{prop})$ (as defined by gender-blind null distribution) could be 0 since we have not guaranteed that $p_{m^{(t)}_{a_1} m^\star_{a_l}} > 0$. In addition, since we are selecting authorships with replacement, $P(X^{prop}) = 0$ if an authorship is selected twice on the cycle. However, if we were to sample without replacement we would need to condition on authorships that had been previously selected, so the proposal probabilities would no longer be symmetric since the probability of traversing a cycle would not be invariant to the orientation of the cycle.

**Remark 1.** *Let $G(X^{(s)} \to X^{(t)})$ be the described proposal distribution in Algorithm 1. Then $G$ is symmetric such that $G(X^{(s)} \to X^{(t)}) = G(X^{(t)} \to X^{(s)})$ and the acceptance ratio of the MCMC-MH procedure only involves the ratio of probabilities in the target distribution.*

*Proof.* Let $X^{(t)}$ and $X^{(s)}$ be two assignments which differ by cycle $\mathcal{A} = \{a_1 \dots a_l\}$. For notational conve-

**Algorithm 1** Proposal Procedure

***Step 1: Sample Cycle***

Select authorship $a_1$ uniformly from $A$

Select authorship $a_2$ uniformly from $\Lambda(f^*_{a_1})$

Draw $l \sim \text{Geometric}(\pi)$

**while** $s < l$ **do**

  $s = s + 1$

  Select authorship $a_z$ uniformly from $\Lambda(f^*_{a_{s-1}})$

**end while**

***Step 2: Generate Proposal with Cycle***

**for** $i \in 1 : (l-1)$ **do**

  $f^{(prop)}_{a_i} = f^{(t)}_{a_{i+1}}$

  $d^{(prop)}_{a_i} = d^{(t)}_{a_{i+1}}$

**end for**

$f^{(prop)}_{a_l} = f^{(t)}_{a_1}$

$d^{(prop)}_{a_l} = d^{(t)}_{a_1}$

***Step 3: Accept or Reject***

$U \sim \text{Unif}(0,1)$

**if** $U < \frac{P(X^{(prop)})}{P(X^{(t)})}$ **then**

  Set $X^{(t+1)} = X^{(prop)}$

**else**

  Set $X^{(t+1)} = X^{(t)}$

**end if**

nience, let $a_0 = a_l$ and $a_{l+1} = a_1$. Then,

$$
\begin{aligned}
G(X^{(t)} \to X^{(s)}) &= \sum_{i\in[l]} G\left(\text{Start cycle at } a_i\right) G\left(\text{Traverse Cycle and end at } a_i | \text{Started at } a_i\right) \\
&= \sum_{i\in[l]} \frac{1}{|A|} G\left(\text{Traverse Cycle and end at } a_i | \text{Started at } a_i\right) \\
&= \sum_{i\in[l]} \frac{1}{|A|} G(a_{i-1} \to a_i) G(a_i \to a_{i+1}) \prod_{j \notin \{i-1,i\}} G(a_j \to a_{j+1}) \\
&= \sum_{i\in[l]} \frac{1}{|A|} (1-\pi) \frac{1}{|\Lambda(f^*_{a_i})|} \prod_{j \notin \{i-1,i\}} \frac{1}{|\Lambda(f^*_{a_j})|} \pi \\
&= \sum_{i\in[l]} \frac{1}{|A|} (1-\pi) \pi^{l-2} \prod_{j \neq i-1} \frac{1}{|\Lambda(f^*_{a_j})|} \\
&= \sum_{i\in[l]} \frac{1}{|A|} (1-\pi) \pi^{l-2} \prod_{j \neq i-1} \frac{1}{|\Lambda(f^*_{a_j})|} \\
&= \sum_{i\in[l]} \frac{1}{|A|} (1-\pi) \pi^{l-2} \prod_{j \neq i} \frac{1}{|\Lambda(f^*_{a_j})|}
\end{aligned} \tag{11}
$$

A proposal of $X^{(t)}$ from $X^{(s)}$ requires traversing the cycle in the opposite direction.

$$\begin{aligned}
G(X^{(s)} \to X^{(t)}) &= \sum_{i\in[l]} G\left(\text{Start cycle at } a_i\right) G\left(\text{Traverse Cycle and end at } a_i|\text{Started at } a_i\right) \\
&= \sum_{i\in[l]} \frac{1}{|A|} G\left(\text{Traverse Cycle and end at } a_i|\text{Started at } a_i\right) \\
&= \sum_{i\in[l]} \frac{1}{|A|} G(a_{i+1} \to a_i) G(a_i \to a_{i-1}) \prod_{j\notin\{i+1,i\}} G(a_j \to a_{j-1}) \\
&= \sum_{i\in[l]} \frac{1}{|A|}(1-\pi)\frac{1}{|\Lambda(f^*_{a_i})|} \prod_{j\notin\{i+1,i\}} \frac{1}{|\Lambda(f^*_{a_j})|}\pi \\
&= \sum_{i\in[l]} \frac{1}{|A|}(1-\pi)\pi^{l-2} \prod_{j\neq i+1} \frac{1}{|\Lambda(f^*_{a_j})|} \\
&= \sum_{i\in[l]} \frac{1}{|A|}(1-\pi)\pi^{l-2} \prod_{j\neq i} \frac{1}{|\Lambda(f^*_{a_j})|}
\end{aligned} \tag{12}$$

□

**Remark 2.** *The Markov chain produced from the proposal procedure in Algorithm 1 is irreducible if* $p_{jk} > 0 \Leftrightarrow p_{kj} > 0$ *and the cycle length* $l$ *is chosen from a distribution with support over* $\{2,\dots|A|\}$ *where* $|A|$ *is the number of authorship instances.*

*Proof.* For each $X^{(s)}$ with $P(X^{(s)}|X^\star) > 0$, there exists a decomposition of $X^{(s)}$ into disjoint sets $\mathcal{A}_1 \dots \mathcal{A}_m$ such that $\mathcal{A}_1$ is a permutation of some subset of $X^\star$. Let $X^{(t)}, X^{(t+1)}, \dots X^{(t+m)} = X^{(s)}$ be the sequence of assignments which correspond to updating the permutation cycles $\mathcal{A}_i$, $i = 1\dots m$. Since there are a finite number of disjoint cycles and the proposal for permuting each cycle is positive, then the joint probability of permuting all cycles is also positive, so $G(X^\star \to X^{(s)}) > 0$. Because the transition support is symmetric, we can also reverse each cycle to move with positive probability from $X^{(t)} \to X^\star$.

Thus, for any two states $X^{(s)}$ and $X^{(t)}$ with positive probability under the null,

$$G(X^{(t)} \to X^{(s)}) \geq G(X^{(t)} \to X^\star)G(X^\star \to X^{(s)}) > 0.$$

□

To allow for collaboration across terminal fields, we use observed citation data from one terminal field to another to define the authorship re-assignment probability, $p_{jk}$, between terminal fields $k$ and $j$. Here, we make two simplifying assumptions. First, we threshold the citation flow between terminal fields at 5% of outgoing citations. Authorship re-assignments between terminal fields that have little connectivity are highly unlikely. Thresholding the citation data produces a network of terminal fields that is sparser (has greater number of disjoint graph components) which allows the sampling procedure to be parallelized more efficiently. Second, to ensure that our sampling procedure can reach all $X^{(t)}$ that have positive probability under the null distribution, we allow for authorship reassignment between terminal fields to be possible in both directions.

More formally, let $p_{jk}$ be observed the proportion of citations from terminal field $j$ to terminal field $k$, $j, k \in F$. We define he authorship re-assignment probabilities between terminal fields as follows:

1. Set any proportions $p_{jk} < .05$ to 0
2. Set $p^*_{jk} = \frac{p_{jk}+p_{jk}}{2}$
3. Renormalize the proportions so $\sum_n p^*_{jk} = 1$

This procedure allows us to take into account substantial connectivity between terminal fields and also ensures that authorship reassignments between terminal fields are possible in both directions:

$$p^*_{jk} > 0 \quad \Leftrightarrow \quad p^*_{kj} > 0.$$

# 6 Results

## 6.1 Sampler Convergence

For the main analysis, we run three different MCMC chains for 150,000 samples each, and combine all three chains (after discarding the first 75,000 as burn-in) to get the final result. To check whether each chain has been run "long enough," we can compare the results from each individual chain. If there is a large discrepancy between the results of any of the individual chains, this would suggest that not enough samples have been gathered. In the figure below, we show the p-value (unadjusted) calculated for each field for Chains 1, 2, and 3. In particular, the horizontal and vertical axis indicate a p-value calculated for a different chain. We would expect the points to lie on the red line with slope 1 if the results from different chains are similar. The top row shows all results, while the bottom row zooms in on the bottom left hand corner and only shows p-values which are close to being statistically significant. Indeed, we see that all dots are near the red line indicating that the results from each individual chain are very similar.

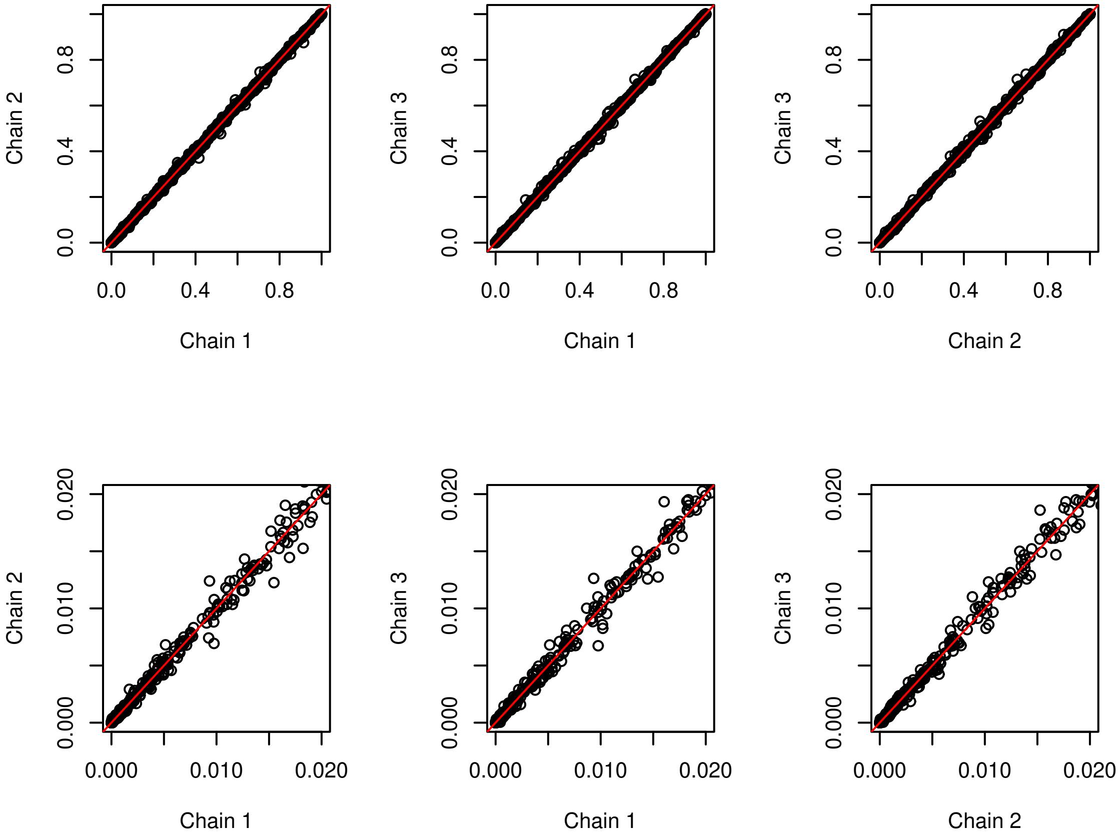


Figure 3: Comparison of p-values generated by each of the three chains.

## 6.2 Comparing observed vs expected $\alpha$

As suggested by a referee, we plot the observed vs expected alpha for each terminal, composite, and top-level field. In the left panel, we include all fields, in the middle panel we only include terminal fields, and in the right panel we only include top and composite fields. As one would expect, we see that there are more points above the 45 degree line than below, indicating that the observed $\alpha$ is typically larger than the expected $\alpha$. This is particularly clear in the panel on the right hand side which only shows results from Top and Composite fields.

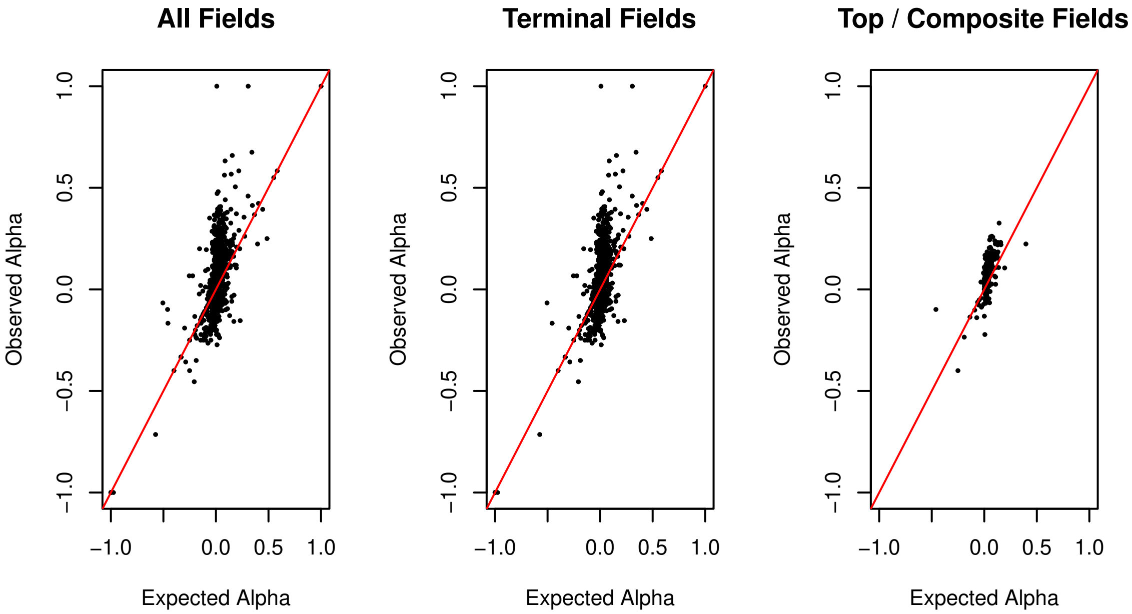

## 6.3 Calculating and adjusting P-values

In the main manuscript, we control the false discovery rate at .05 with the Benjamini-Yekutieli procedure (Benjamini and Yekutieli, 2001) which allows for arbitrary dependence of the p-values, but is more conservative than the Benjamini-Hochberg procedure (Benjamini and Hochberg, 1995), which only allows for certain types of positive dependence. Table 4 replicates Table 1 of the main manuscript using the Benjamini-Hochberg procedure (instead of Benjamini-Yekutieli) with FDR rates of .05 and .005.

Table 4: **Main results under Benjamini-Hochberg.** Results for the JSTOR corpus and each top-level field, sorted largest to smallest (top to bottom) by number of authorships. The $\alpha$ column gives the observed value and expected value under no behavioral homophily; the $WM$ column gives the number of heterophilous documents corresponding to the observed and expected $\alpha$. The "P-value" column gives the Benjamini-Hochberg adjusted p-value for the top-level field; "Terminal" and "Composite" columns give the counts of terminal and composite fields are which significant at the .005 level / significant at the .05 level / total.

| Field | Observed / Expected | | P-value | Sig .005 / Sig .05 / Total | |
|---|---|---|---|---|---|
| | $\alpha$ | $WM$ | | Terminal | Composite |
| JSTOR | .11/.06 | 34/35.7 | 0 | 63/167/1450 | 48/89/280 |
| Mol/Cell Bio | .05/.02 | 38.2/39.4 | .00 | 12/40/178 | 14/22/44 |
| Eco/Evol | .06/.04 | 31.9/32.8 | .00 | 7/16/257 | 7/14/56 |
| Economics | .11/.04 | 18.7/20.3 | .00 | 5/17/136 | 5/12/28 |
| Sociology | .19/.10 | 38.5/42.9 | .00 | 8/19/94 | 8/13/21 |
| Prob/Stat | .09/.05 | 26/27.3 | .00 | 0/3/90 | 0/3/23 |
| Org/mkt | .16/.06 | 29/32.5 | .00 | 6/12/68 | 3/3/4 |
| Education | .16/.07 | 41.2/45.8 | .00 | 5/14/42 | 4/5/10 |
| Occ Health | .10/.03 | 41.7/44.9 | .00 | 7/12/24 | 1/1/1 |
| Anthro | .12/.07 | 38.5/40.7 | .00 | 1/5/63 | 0/1/8 |
| Law | .17/.11 | 29.7/31.7 | .00 | 0/1/98 | 0/0/16 |
| History | .16/.09 | 32.9/35.6 | .00 | 0/1/49 | 0/1/6 |
| Phys Anthro | .07/.02 | 34.7/36.3 | .00 | 1/4/32 | 2/3/10 |
| Intl Poli Sci | .09/.04 | 27.3/28.7 | .04 | 0/0/34 | 0/0/2 |
| US Poli Sci | .15/.10 | 25.2/26.7 | .01 | 1/1/37 | 0/3/6 |
| Philosophy | .10/.06 | 18.7/19.6 | .10 | 0/2/45 | 0/0/8 |
| Math | .04/.03 | 14.3/14.4 | .53 | 0/1/46 | 0/0/9 |
| Vet Med | .09/.03 | 38.3/40.6 | .00 | 4/7/19 | 0/1/2 |
| Cog Sci | .18/.11 | 35.7/38.7 | .00 | 3/5/14 | 3/3/3 |
| Radiation | .09/.03 | 34/36.3 | .00 | 3/4/14 | 1/3/5 |
| Demography | .15/.08 | 40.3/43.3 | .00 | 0/1/20 | 0/1/2 |
| Classics | .07/.03 | 38.7/40.6 | .10 | 0/0/35 | 0/0/8 |
| Opr Res | .03/.03 | 16.6/16.7 | .59 | 0/0/18 | 0/0/4 |
| Plant Phys | .08/.05 | 29.1/30.2 | .10 | 0/2/21 | 0/0/3 |
| Mycology | .03/.03 | 36.8/36.7 | .79 | 0/0/16 | 0/0/1 |

### 6.4 Secondary Analysis

In the main text, we examined whether certain terminal field characteristics are associated with statistically significant behavioral homophily. In particular, we fit a logistic regression where the dependent variable is whether or not significant behavioral homophily was detected using the Benjamini-Yekutieli FDR procedure (Benjamini and Yekutieli, 2001) with a cut-off of .05. We fit the logistic regression using a generalized estimating equation (GEE) (Vincent J Carey and Ripley, 2015); to account for dependency across terminal fields, we use robust standard errors and specify clusters aligning to top level field. We also specify a diagonal working covariance.

Below, we also show the results when using different cut-offs and FDR procedures.

Table 5: Results of the logistic regression using significant behavioral homophily under the Benjamini-Yekutieli FDR procedure with a .005 cut-off as the dependent variable.

| | Estimate | Robust S.E. | Robust z | P-value |
|---|---|---|---|---|
| Intercept | -19.25 | 2.27 | -8.50 | 0.00 |
| log(Authorships) | 1.78 | 0.26 | 6.75 | 0.00 |
| Proportion women | 10.82 | 2.13 | 5.09 | 0.00 |
| Majority women indicator | 8.36 | 7.57 | 1.10 | 0.27 |
| Ratio Solo vs Multi women | -0.17 | 0.50 | -0.34 | 0.73 |
| Proportion women × Majority women interaction | -15.75 | 12.96 | -1.22 | 0.22 |

Table 6: Results of the logistic regression using significant behavioral homophily under the Benjamini-Hochberg FDR procedure with .05 cut-off as the dependent variable.

| | Estimate | Robust S.E. | Robust z | P-value |
|---|---|---|---|---|
| Intercept | -12.29 | 1.18 | -10.42 | 0.00 |
| log(Authorships) | 1.20 | 0.13 | 9.32 | 0.00 |
| Proportion women | 7.88 | 1.44 | 5.47 | 0.00 |
| Majority women oindicator | 10.27 | 5.56 | 1.85 | 0.07 |
| Ratio solo vs multi women | -0.01 | 0.26 | -0.05 | 0.96 |
| Proportion women × Majority women interaction | -19.46 | 9.74 | -2.00 | 0.05 |

Table 7: Results of the logistic regression using significant behavioral homophily under the Benjamini-Hochberg FDR procedure with .005 cut-off as the dependent variable.

| | Estimate | Robust S.E. | Robust z | P-value |
|---|---|---|---|---|
| (Intercept) | -15.10 | 1.53 | -9.85 | 0.00 |
| log(Authorships) | 1.44 | 0.20 | 7.22 | 0.00 |
| Proportion women | 7.46 | 1.33 | 5.62 | 0.00 |
| Majority women indicator | 10.47 | 5.14 | 2.04 | 0.04 |
| Ratio solo vs multi women | 0.06 | 0.43 | 0.15 | 0.88 |
| Proportion women × Majority women interaction | -19.15 | 9.09 | -2.11 | 0.04 |

## 6.5 Sensitivity Analysis: Missing Gender Indicators

To evaluate how sensitive our main results are to the missing gender indicators, we impute gender for authorships with missing gender indicators under two scenarios:

- Low homophily: Each authorship with a missing gender indicator is assigned a gender at random according to the proportions of observed genders on its original terminal field. This procedure assumes that there is no behavioral homophily in the imputed data because the imputed genders are conditionally independent given the terminal field. Thus, it gives a reasonable lower bound on the homophily we might have observed given the full data.

- High homophily: Each authorship with a missing gender indicator is assigned a gender at random according to the proportions of observed genders on its original paper. If the original paper contains only authorships with missing gender indicators, we assign all authorships on the paper the same gender indicator which is drawn randomly according to the proportions of observed genders for its original terminal field. Because papers with at most one assigned gender indicator are homophilous by construction, this provides a reasonable upper bound on the homophily we might have observed given the full data.

For each scenario, we carry out 10 imputations and then repeat the entire sampling and testing procedures used for the main analysis. Table 8 gives the resulting percentages of terminal, composite, and top level fields with significant behavioral homophily under the Benjamini-Yekutieli FDR procedure with $\alpha = .05$ under the low and high homophily missing data imputation scenarios. We observed that, on average, $.04, .15, .66$ of terminal, composite, and top level fields exhibit statistically significant respectively in the low homophily scenario; for the high homophily procedure the corresponding averages are $.48, .79, 1.00$.

Table 8: Each column shows the percentage of fields which exhibit statistically significant homophily for each of the individual imputations

| | Terminal | Composite | Top |
|---|---|---|---|
| Main Analysis | 0.05 | 0.18 | 0.71 |
| Low Imputation 1 | 0.04 | 0.13 | 0.67 |
| Low Imputation 2 | 0.04 | 0.17 | 0.62 |
| Low Imputation 3 | 0.03 | 0.15 | 0.67 |
| Low Imputation 4 | 0.04 | 0.16 | 0.67 |
| Low Imputation 5 | 0.03 | 0.14 | 0.67 |
| Low Imputation 6 | 0.04 | 0.17 | 0.67 |
| Low Imputation 7 | 0.04 | 0.17 | 0.67 |
| Low Imputation 8 | 0.04 | 0.16 | 0.67 |
| Low Imputation 9 | 0.04 | 0.15 | 0.67 |
| Low Imputation 10 | 0.04 | 0.16 | 0.62 |
| Low Imputation Avg | 0.04 | 0.15 | 0.66 |
| High Imputation 1 | 0.48 | 0.80 | 1.00 |
| High Imputation 2 | 0.48 | 0.78 | 1.00 |
| High Imputation 3 | 0.48 | 0.79 | 1.00 |
| High Imputation 4 | 0.48 | 0.79 | 1.00 |
| High Imputation 5 | 0.48 | 0.79 | 1.00 |
| High Imputation 6 | 0.47 | 0.79 | 1.00 |
| High Imputation 7 | 0.48 | 0.79 | 1.00 |
| High Imputation 8 | 0.48 | 0.80 | 1.00 |
| High Imputation 9 | 0.48 | 0.80 | 1.00 |
| High Imputation 10 | 0.48 | 0.79 | 1.00 |
| High Imputation Avg | 0.48 | 0.79 | 1.00 |

# References


Benjamini, Y. and Hochberg, Y. (1995). Controlling the false discovery rate: a practical and powerful approach to multiple testing. *Journal of the royal statistical society. Series B (Methodological)*, pages 289–300.

Benjamini, Y. and Yekutieli, D. (2001). The control of the false discovery rate in multiple testing under dependency. *Ann. Statist.*, 29(4):1165–1188.

Larivière, V., Ni, C., Gingras, Y., Cronin, B., and Sugimoto, C. R. (2013). Bibliometrics: Global gender disparities in science. *Nature News*, 504(7479):211.

Ridout, M. S., Demétrio, C. G. B., and Firth, D. (1999). Estimating intraclass correlation for binary data. *Biometrics*, 55(1):137–148.

Vincent J Carey, T. L. and Ripley, B. (2015). *gee: Generalized Estimation Equation Solver.* R package version 4.13-19.
