# Supplementary material for: Gender-based homophily in collaborations across a heterogeneous scholarly landscape
Source: PLoS One. 2023 Apr 5;18(4):e0283106. doi: 10.1371/journal.pone.0283106 (PMC10075399; doi:10.1371/journal.pone.0283106)
Supplement: Attachment — Submitted filename: responses .pdf [file pone.0283106.s002.pdf]

Reviewer Responses

We would like to thank the reviewers and AE for the very helpful feedback in improving the manuscript. We have substantially revised the manuscript and analysis as a result. Below, we have summarized the major changes and have also included a point-by-point response to the reviewer comments.

Major Updates

- Most notably, at the suggestion of Reviewers 1 and 2, we now explicitly account for publication year in our analysis. In particular, the null distribution now includes a term which drastically upweights configurations where authorships are permuted to other documents whose publication dates are similar to the publication date of the authorship's original document. As expected, this decreases the number of fields with significant behavioral homophily. Nonetheless, a substantial number of fields still exhibit behavioral homophily even after accounting for publication year in our analyses.
- At the suggestion of Reviewer 2, we have restructured our paper into the following sections: Introduction, Problem Setup, Data, Methods, Results, and Discussion
- In response to concerns from both Reviewer 1 and 2, we've weakened the claim we make. Specifically, the document now says that we test whether "the observed gender homophily can be fully explained by varying gender composition across intellectual communities and time" as opposed to what we had originally written about testing "whether gender has a direct effect on co-authorship formation." Thus, we do not explicitly rule out confounding due to race, ethnicity, or career levels, though we included some discussion of this in Section 6.
- In the Methods section, we have substantially re-written the "Homophily in a heterogenous setting" to better explain our approach. In particular, we further expound on the example which highlights the way that we can test for behavioral homophily in a top/composite field but still account for the compositional homophily induced by terminal field structure. In addition, our previous discussion of structural and compositional homophily only explicitly mentioned how they may change the expected value of a distribution for alpha. We now explicitly mention that they change not only the expected value but the shape of the distribution as well.

Reviewer 1

1. ***The paper analyses the degree of gender homophily in collaboration patterns among JSTOR articles. As the authors argue, empirical patterns indicating gender homophily can arise due to patterns and mechanisms on different levels. In particular, different gender distributions in small subfields may produce homophily in composite fields as well as behavioral homophily on the individual level. The authors provide an interesting methodological approach for distinguishing between these different sources of homophily by assessing the degree of homophily at different levels of field-classifications. However, the authors should consider the following points in a revised version before publication.***

2. ***36-40: The reference for the "Wahlund effect" should already be included here***

   - We have updated the manuscript to include the reference.

3. ***57-60: Other reasons besides "cultural norms" may also play a role here. Especially different career levels for female and male researchers. This could also be mentioned in line 151.***

- We have added discussion on different career levels to the discussion section.

4. ***67-68: The fact that there are many algorithms for author name disambiguation is not sufficient for arguing that disambiguated data would be of poor quality. One should explicitly argue that the quality that existing disambiguation approaches produce is of too poor quality for the analyses.***

    - Tekles and Bornmann (2020) compare various author disambiguation procedures against a baseline which uses initials and surnames. When considering the task of assigning an authorship to a single author, they find that the only procedure which substantially outperforms the baseline (Table 1 $F1_{best}$) is a procedure by Caron and van Eck (2014) which scores an author match based on various metadata characteristics. In their scoring rule, the feature which has the strongest affect on score is author email address. Unfortunately, because we consider data from 1960-2011, for the vast majority of our data, the email addresses of the authors are not available so its not clear that we would be able to do much better than the baseline disambiguation procedures.

      Furthermore, Kim and Diesner (2016) show that a simple disambiguation procedure using initials and surname can severely bias the results when measuring individual level properties of collaboration networks. In particular, they compare results using several disambiguation procedures using initials and surnames to results using a proxy for ground truth. The procedure to create the proxy ground truth uses full name, names of coauthors, affiliation information, and email address. Kim and Diesner find that using procedures based on initials and surnames distorts many measured properties of the collaboration network including network density, author productivity, clustering coefficients, and—most relevant to our setting—assortativity. In particular, all of the simple procedures they consider—when averaged across several data sets—decrease measures of assortativity by at least 25%. Kim (2019) shows that the "scale-free-ness" of collaboration networks can also be drastically biased when using a simple disambiguation procedure.

      Thus, we use authorships rather than authors. This more agnostic approach clearly presents the limitations of our data as opposed to using poorly disambiguated data which likely overstate our level of data quality.

      Caron, E., & van Eck, N. J. (2014). Large scale author name disambiguation using rule-based scoring and clustering. In E.Noyons (Ed.), Proceedings of the Science and Technology Indicators Conference 2014 Leiden (pp. 79–86). Leiden: Universiteit Leiden—CWTS.

      Kim, J., & Diesner, J. (2016). Distortive effects of initial-based name disambiguation on measurements of large-scale coauthorship networks. Journal of the Association for Information Science and Technology, 67(6), 1446-1461.

      Kim, J. (2019). Scale-free collaboration networks: An author name disambiguation perspective. Journal of the Association for Information Science and Technology, 70(7), 685-700.

      Tekles, Alexander, and Lutz Bornmann. "Author name disambiguation of bibliometric data: A comparison of several unsupervised approaches." Quantitative Science Studies 1.4 (2020): 1510-1528.

5. ***71: One sentence on how alpha should be interpreted (which range? what do large/small values mean?) could help the reader to get an idea of how this indicator works?***

- In the second and third paragraph of the “Quantifying homophily” section, we now begin with a very broad description of α before giving additional details in the remainder of the paragraph.

6. **91: It is not obvious why alpha = -1/(2-2pi) if the proportion of female-male papers is 2pi. A derivation of this equation may be helpful to understand this calculation.**

    - Thanks for pointing this out. There is actually a small typo where α = -pi/(1-pi). We have given additional details in the supplement, and indicated in the main text that “Details on these calculations are given in the supplement.”

7. **97-104: It is not obvious why there should be structural homophily as defined in this manuscript. If only the number of authorships, the number of co-authors, the number of female authors and the number of male authors are fixed, why should the probability that a randomly selected co-author is male differ between a female and a male authorship? If this should be the case, then the gender of a co-author somehow needs to depend on the gender of a given authorship. However, this is not reflected in the aforementioned assumptions (unless there are some correlations among the fixed parameters?). It would be helpful to clarify the notion structural homophily and/or clarify how it emerges under the assumptions. See also the example in the remark for lines 262-263.**

    - We have expanded the discussion of structural homophily in Section 2.2 to be more clear for the reader. Structural homophily reflects the fact that even when authorships are being permuted at random, the distribution of alpha depends on “structural characteristics” of the field (previously we had only explicitly discussed differences in the expected value due to structural characteristics, but we have now explicitly mentioned changes in the shape of the distribution as well). This is because there are a finite number of authorship configurations, and the possible values of alpha (as well as the probability for each of those possible values) depends on the number of authorships of each gender as well as the number of authorships on each paper. In Figure 2 of the main text, we show the distributions of sub-field A and B when accounting for structural characteristics. As can be seen in the top panels, the two distributions differ drastically.

8. **128: This sentence seems a bit unclear, the authors may want to reformulate it.**

    - We have updated the sentence to “In contrast to structural and compositional homophily which could occur when co-authors are selected irrespective of gender, we use *behavioral homophily* to describe deviations of α from its distribution under structural and compositional homophily.”

9. **175-176: "... which were clustered into one of the terminal fields ...": Are there papers that were not clustered in any of the terminal fields? If so, how many?**

    - We have now clarified at the end of Section 4 that “*243,945 papers and 755,913 authorships are removed because they are not clustered into a terminal field*”

10. **183-184: "all relevant aspects of co-authorship choice except behavioral homophily" -> This is in contrast to what is mentioned in line 151 (there are some factors that cannot be included). I think this should also be mentioned here.**

- We have updated the text to make a weaker claim, and have added discussion to include additional sources of compositional homophily. We now make it clear that we test the null hypothesis that the observed gender homophily may be fully explained by the structural and compositional homophily (with respect to sub-fields and time) which we model. We now explicitly note that behavioral homophily may include explicit consideration of gender, but also may be caused by other correlated factors such as race, ethnicity, and career stage.

11. **193-202: Are inter-field swaps really necessary here? The fixed distribution parameters are based on a situation where inter-field swaps have already occurred, i.e. the observed gender distribution, no. of co-authors etc. already account for inter-field swaps.**

    - Inter-field swaps are not strictly necessary. In fact, the earliest iteration of our analysis did not involve inter-field swaps and the sampling procedure becomes much less complicated when they are not allowed. However, we include them in our testing procedure because they soften the impact of the “discrete” nature of the hierarchical clustering. We have included text in Section 4.1 to explain “However, we still allow swaps between nearby terminal-field with small probability to reflect cross-field collaborations. This also makes the null distribution less sensitive to the otherwise discrete clustering of documents to terminal fields.”

12. **209: "... for each configuration to form q distribution ..." -> What means "q" here?**

    - This was a typo and “q” should be “a”

13. **209-219: Why is it necessary / what is the benefit to implement your sampling procedure instead of simply using the share of female (male) authorships (inter-field swaps could be included based on the share of female (male) authorships in the other fields) as probabilities for assigning the gender to the fixed paper-author instances (and then calculating alpha based on the resulting configuration)?**

- There are important differences between fixing the set of authorships and permuting them to different papers as opposed to re-sampling a new set of authorships where the genders of each authorship are drawn from some fixed distribution. In our testing procedure, we choose a finite-population approach which permutes the observed set of authorships rather than an infinite-population approach which repeatedly resamples a completely new set of authorships. The finite-population approach—which we take—makes inferential statements conditional on the documents, authorships, and heterogeneous gender representation which we actually observed. We choose this approach because it allows us to test our hypothesis in an “assumption lean" way, while the infinite-population approach would require assuming a specific generative procedure for authorships. In addition, the infinite-population approach puts larger probability on configurations where a field consists of authorships of only one gender and alpha is undefined. When the field is small and/or the proportion of women is very close to 1 or 0, the probability that alpha is undefined can be quite large. For example, in Field B in Fig~1, if we resample authorships according to the observed gender proportions, this occurs with probability roughly 1/3. We briefly discuss this in Section 4.1.

14. **234: It is not obvious how the formula for calculating the number of heterophilous papers is derived. Adding this information may be helpful for the reader.**

- We have given additional details in the supplement, and indicated in the main text that "details of the calculations are given in the supplement."

15. **262-263: If authorships are assigned randomly and there is no homophily within two subfields with different gender distributions, taken together there is a certain degree of homophily to be expected (assuming that there is more collaboration within the subfields than between them). Consider the following example: subfield A with 100 female authorships and 900 male authorships, subfield B with 900 male authorships and 100 female authorships. If the authorships are randomly (with constant probability) assigned to the given paper-author instances, a given authorship has a probability of 90% in subfield A and 10% in subfield B that a randomly selected co-author is male. This is true irrespective of the gender of the given authorship (because the authorship of the co-author has been drawn from the same distribution, irrespective of the gender of the given authorships). Thus, alpha is 0 in both subfields. Assuming that there is no collaboration between the subfields, the share of female authorships from subfield A (s_af) among all female authorships is .1 (for subfield B: s_bf = .9), and the share of male authorships from subfield A (s_am) among all male authorships is .9 (for subfield B: s_bm = .1). Thus, for the composite field containing both subfields, alpha = p - q = (s_am * .9 + s_bm * .1) - (s_af * .9 + s_bf * .1) = (.9 * .9 + .1 * .1) - (.1 * .9 + .9 * .1) = .82 - .18 = .64. Of course this value reduces if there are collaborations between the two fields, but it seems reasonable to expect more collaboration within the fields than between them. This means that the homophily in composite fields inevitably increases compared to the subfield within the composite field. I would assume that this effect is more important than the increasing testing power when looking at composite fields (only mentioning testing power here somehow undermines the authors key contribution of distinguishing different forms of homophily, in particular composite homophily vs. behavioral homophily). Further elaborating on this point could help to better understand the relationship between compositional homophily and behavioral homophily.**

    - The example you describe is exactly the compositional homophily phenomenon which we adjust for. As you mention, when multiple sub-fields with differing proportions of man and woman authorships are measured in aggregate, we would expect the observed value of alpha to be positive even when co-authorships are randomly assigned. However, we account for the sub-field structure in the null distribution which reflects the compositional homophily, so the null distribution (which we compare the observed statistic against) will also produce generally larger values of alpha. Thus, detecting statistically significant behavioral homophily suggests that the observed alpha is large--even beyond the compositional homophily. Thus, it is critical not just to view the observed value of alpha, but to also compare it to the null distribution. We have substantially re-written Section 2.2 to describe our approach more clearly.

16. **273-274: Adding the percentages (analogous to 255-256) would be helpful to compare the results for both approaches.**

    - This has been updated in the manuscript

17. **408-412: Restricting the sample to papers published no earlier than 1960 seems to be a very weak restriction. Thus, the mitigating effect of this restriction may be neglectable. An alternative robustness check would be to further restrict the sample to papers published**

in more recent years and test whether there are great differences compared to the main results.

- We have now explicitly encoded a temporal element into our null distribution. Under the null distribution, the probability of each permutation of authorships to documents now depends not only on the original/currently assigned terminal field of each authorship, but also the publication year of the original/currently assigned document. Specifically, we now include an additional term which makes it much more likely for authorships to be reassigned to documents which are close in publication year. This is described in detail in Section 4 of the main manuscript. Explicitly accounting for publication year does indeed reduce the number of fields with significant homophily, as would be expected. However, the majority of top level fields and many composite fields still exhibit significant behavioral homophily.

18. **425: Should "[?]" be a reference?**
   - Yes. This has been updated in the manuscript
19. **357: "with with" -> "with"**
   - This has been updated in the manuscript
20. **400: "but can controlled" -> should this be "but can be controlled"?**
   - This has been updated in the manuscript
21. **Page 14, last paragraph: "Larivière hand-check a sample of 1000..." -> Should "Larivière" be a reference?**
   - This has been updated in the manuscript
22. **page 22: The upper bounds for the homophily in the sensitivity analyses are very high. An interpretation of this result (especially in contrast to the small values for the main result and the lower bound in the sensitivity analyses) would be helpful to put this in context.**
   - We have added the following discussion to Section 5.3:

     As can be seen in Table 3, compared to the main analysis, the low homophily setting indeed has a lower proportion of fields with significant behavioral homophily, and the high homophily setting has a higher proportion of fields with significant behavioral homophily. However, while the results of the low homophily analysis do not drastically differ from the main analysis, the results of the high homophily analysis are substantially higher than the main analysis. We posit two potential reasons for this. First, we previously did not include authorships for which we did not estimate a gender; thus, the sensitivity analysis actually increases our “sample size” and may increase power to reject the null hypothesis. Thus, although we would expect to detect fewer fields with significant homophily in the low homophily setting, this may be partially counteracted by the increased power. Second, both strategies generally seek to impute authorship gender with no behavioral homophily. However, when a document has one or less estimated authorships, the high homophily strategy imputes genders in a directly homophilous way; this is in contrast to the low

homophily strategy which does not employ an imputation strategy which is explicitly heterophilous.

Reviewer 2

1. **Reviewer #2: The authors analyze "gender based homophily" in co-authorships: Do men and women tend to collaborate in particular with coauthors of the same gender, and does this still hold when one accounts for structural and composition effects, that exist e.g. with the different distribution of male and female authors across different research fields and topics (authors call the latter "intellectual interests"). This is certainly an interesting topic; there is much speculation to what extent gendered preferences or structural barriers contribute to gendered patterns in publications and related success in science. Authors use large-N data (data on all publications published in JSTOR in 1960-2011), and they use innovative methods to identify research topics (intellectual interests) by cluster methods. They also provide many robustness checks. However, according to my impression, there are also strong limitations, which I will explain in the following.**

2. **Clarity of the argumentation: This is my main issue. I am not an expert in the applied cluster approach and statistical measures, but I am at least familiar with theories on homophily. For me, it was nevertheless very difficult to follow the line of arguments. I would recommend to explain the assumed mechanisms more clearly – for instance, why is there structural homophily in addition to compositional homophily? What makes the difference? One could start with explaining more explicitly what is the difference between "fields" (underlying Fig 1 to explain "structural" homophily) and "intellectual interests", which are also defined by different "fields" – labeled top-level, compositional and terminal fields. This is confusing. I would also recommend that you stick mainly to ONE terminology to explain the different sources for homophily, and to use it from the beginning on (e.g. in the introduction, there is reference to the "Hardy-Weinberg equilibrium"; only readers from the field of genetics will be able to understand this). And to give another example: Currently, the reader has to figure out what the labels for the different sensitivity analyses in Table 3 mean (they are not introduced in the text where the sensitivity analyses are explained). Those are only few examples. I had to re-read several sections to grasp the meaning.**

    - Thanks for the helpful comments. We have substantially revised the entire document to clarify our arguments and methodological contributions.

      In particular, to more clearly explain our approach in Section 2, we first clarify that "observed gender homophily" indicates the observed value of alpha---regardless of the process under which authorships formed. We have also made it more explicit that the structural and compositional homophily refers to mechanisms which affect the distribution of alpha, which are tied to, but not exactly, mechanisms which may be intrinsic to the co-authorship formation process. We also give examples of structural and compositional homophily to build intuition.

      We have retained the discussion on other fields in which this type of phenomenon is studied because we think it is important to connect our work to previous work and point our readers towards a rich literature. We also now explicitly note the labels for the

sensitivity analysis.

3. **To improve clarity of argumentation, it would also help to structure the article more clearly into theory, methods, data, result. For example, in the current version, theories on possible mechanisms are included in the "result section."**
    - We have restructured the manuscript into a form which roughly mirrors the suggestion. In particular, we have restructured/re-ordered our paper into the following sections: Introduction, Problem Setup, Data, Methods, Results, and Discussion
4. **What is also confusing is to speak of "behavioral homophily" when this kind of homophily is probably again caused by composition effects (authors themselves indicate e.g. race as a candidate for such composition effects). Even more important: The only vague mechanisms authors mention for this kind of homophily are "cultural norms" – could you explain what you mean by that? HomoPHILY is, to my understanding, typically associated with preferences (from Ancient Greek: phily = friendship/love). These preferences might be caused by social norms, but anyway: it would be good to explain.**
    - In section 2, we have now included text to mention specific alternative explanations such as race, ethnicity, and career level. We also include a more extended discussion in the Discussion section.

      In particular, in assessing whether the unobserved factors invalidate our results, we note that not all homophily with respect to a certain characteristic may induce compositional homophily (with respect to gender). Specifically, to induce compositional gender homophily, the characteristic must be associated both with co-authorship probability and authorship gender.
5. **I was also wondering why you don't include the year of publication into the reference distribution (measuring the baseline homophily without behavioral homophily). With more and more women entering science over time, the behavioral homophily probably mostly measures this time trend. (One might simply run robustness analyses pooling not all papers that appeared within 50 years, but only papers within a time frame where it is more likely that researchers are active at the same time and therefore can in principle be coauthors).**
    - We have now explicitly encoded a temporal element into our null distribution. Under the null distribution, the probability of each permutation of authorships to documents now depends not only on the original/currently assigned terminal field of each authorship, but also the publication year of the original/currently assigned document. Specifically, we now include an additional term which makes it much more likely for authorships to be reassigned to documents which are close in publication year. This is described in detail in Section 4 of the main manuscript. Explicitly accounting for publication year does indeed reduce the number of fields with significant homophily, as would be expected. However, the majority of top level fields and many composite fields still exhibit significant behavioral homophily.
6. **Are there really problems with statistical power with these large N data? I would like to see some more information on power analyses to get convinced. If only the "top level" fields are used, one controls for "intellectual interests"/"research topics" only with very broad**

research disciplines (such as Economics versus Law versus History). I am not convinced that this helps to net out the compositional homophily. It is well known that even within disciplines men and women cluster into different subfields.

- As you rightly point out, one would expect compositional effects within "top level" fields to cause a larger value of alpha, even if no behavioral homophily is present. Indeed, our main methodological innovation is accounting for this effect. When testing the "top level" or "composite" fields, we crucially do not permute all authorships uniformly, but only permute authorships within their terminal field (or within "nearby" terminal fields). Thus, the null distribution to which we compare the observed statistic, adequately reflects the compositional effects induced by sub-field heterogeneity. We believe that the terminal fields we use are narrow enough to characterize intellectual interests. In the time period we consider (1960-2011), the median number of total documents in each terminal field is 102 (roughly 2 documents a year), and 95% of terminal fields had less than 570 total documents (roughly 11 documents each year). Thus, the values of alpha from the null distribution, even when measured on a top level field, do capture compositional homophily induced by sub-field structure. We have revised Sections 2 and 4 substantially to better explain our approach. In addition, we now give a more detailed example in Fig 2, where the null distribution captures compositional effects when aggregating two sub-fields.

7. **In the "Main Analysis", the authors introduce some additional measures. I strongly recommend to move the explanation of these measures to a method section, where all different measures are explained (with their respective advantages/shortcomings). For me it was not easy to understand why it is helpful to have in addition this FM measure.**
    - We have moved the definition of this metric to Section 2.1 and added text to better explain the purpose of the measure. In short, the units of alpha are "probabilities" but the practical impact of gender homophily (as measured by potential heterophilous teams) is also dependent on the gender representation within each field. Thus, we use the FM as a concrete measure of impact.
8. **Results could be nicely presented in a Figure, where one plots observed and expected alphas against each other, together with a reference line at 45° that would indicate that both converge.**
    - We have included the suggested plot in the supplement section 6.2. We plot the expected alpha on the horizontal axis and the observed alpha on the vertical axis. As one would expect, we see that there are more points above the 45 degree line than below, indicating that the observed alpha is typically larger than the expected alpha. This is particularly clear in the panel on the right hand side which only shows results from Top and Composite fields.
9. **Why do you use for the "Secondary Analysis" a binary outcome variable? As discussed by the authors, definitions of "significance" are always somewhat arbitrary. Why don't you use instead a metric variable, such as the ratio of observed to expected alpha values. I am not completely sure whether this really makes sense, but one might at least discuss the possibility of using such an alternative (and probably more informative) metric.**
    - We agree that using the binary outcome of significance may not be perfect; however, as

we show the qualitative results of the secondary analysis are not sensitive to the significance level cut-off. The biggest benefit of using the binary outcome of significance vs non-significance is that it ensures that the analysis is well connected to the primary metric we report throughout the paper: significant vs non-significant behavioral homophily.

The ratio of observed to expected alpha value's becomes very unstable when the expected values of alpha are close to zero (as is often the case), and a negative ratio becomes very hard to interpret (e.g., -.1 / .1 is very different than -.5 / .5 but otherwise would appear the same in our analysis). One could also look at the difference between observed and expected, but this only compares the observed alpha to the mean of the null distribution and does not account for the variance of the distribution, which is critical for interpreting the results.

One potential alternative metric would be the "z-score" for each field where we take (alpha_obs - alpha_exp) / sigma_alpha where alpha_obs is the observed value of alpha, alpha_exp is the expected value of alpha under the null distribution, and sigma_alpha is the standard deviation of alpha under the null distribution. Ultimately, however, the results of the regression become difficult to interpret concretely since the z-score is only loosely tied to our outcome of interest.

10. **Providing more consistent information on all methods would be helpful. For instance, I missed information on the number of "compositional fields". (There seems to be only information on the number of top level and terminal fields)**
    - The number of compositional fields (both in total and for each top level field) are included in Table 1. In general, we have included additional text on the methods in Section 4 to better explain our approach.
11. **To sum up: The authors work on an interesting research topic, providing some novel methods to define different research topics/intellectual interests. However, the current version of the manuscript is very difficult to understand. One might easily improve the writing style, but I am also not very convinced that the methods and results authors provide are really helpful: The main idea to remove compositional effects would require that one is really able to identify the "intellectual interests." This might be achieved fairly well with the terminal fields, but according to the authors, these more narrow fields miss statistical power. And even within these more narrow fields, gender is still likely confounded with research topics (that might be even more specific) and a lot of other structural/compositional factors. So, what we can probably only conclude from this paper is that broad research disciplines are not the only explanation for "gender homophily." Given the fact that authors do not even exclude publication year as an alternative explanation, I found the discussion on possible discrimination, stereotype threat effects etc. in the discussion much too speculative. Anyway, I hope that my review is helpful.**
    - Thanks for the many helpful comments. We agree that conditioning on broad research topics is not adequate to adjust for heterogeneity by "intellectual interests" and indeed our methodology is motivated by that inadequacy. Thus, our analysis explicitly accounts for terminal fields which we believe adequately captures the very fine-grained structure of "intellectual interests" (the median terminal field is 102 documents which amounts to

roughly 2 documents a year). We have substantially rewritten the manuscript to more clearly explain how we account for the terminal field structure even when considering the larger composite and top-level fields. In addition, we have revised our analysis to account for temporal heterogeneity. In our discussion throughout the paper, we also include explicit discussion of alternative explanations and have weakened the claims we make about gender discrimination.
