# Supplementary material for: Gender-based homophily in collaborations across a heterogeneous scholarly landscape
Source: PLoS One. 2023 Apr 5;18(4):e0283106. doi: 10.1371/journal.pone.0283106 (PMC10075399; doi:10.1371/journal.pone.0283106)
Supplement: Attachment — Submitted filename: responses.pdf [file pone.0283106.s003.pdf]

Reviewer Responses

- *The paper resorts to the JSTOR database and then (in lines 599 to 601) suggests that it could be employed to analyze gender homphily in other collections (such as WoS or Scopus). I am not that familiar with JSTOR database myself and this is not a very popular source for bibliometric analysis. It remains a innovative feature of this research to resort to such a data source. Still, every database has particular features that limit the reach and scope of the analysis conducted using its data. I wonder if some clarification on the reasons for choosing this database for the study would help further research. The accounts given –in lines 46 to 48; and 250 to 252— do not state any strengths and weaknesses of this data source. If this research is to be continued using input from other databases, it would be useful to know the grounds in which this data source has been selected*

  The JSTOR database includes a wide variety of journals across various disciplines; however, as you note, other databases may be more popular for bibliometric analysis. We choose to use JSTOR for our analysis because previous work has developed a heirarchical clustering of the JSTOR corpus based on citation networks. This yields a much finer representation of the scientific landscape as opposed to simply clustering by journal. This heirarchical clustering is crucial to our analysis. In addition, each identified cluster has been painstakingly hand labeled; this allows for interpretable results which are also useful for generating hypotheses for future work. We have added text in Section 3 to explain.

- *Some repetitions appear in the manuscript, a careful check could be useful. For instance, in page 2, it repeats "the the" (line 54) and "might might" (line 57).*

  Thank you for comment. We have carefully read through the manuscript to remove the typos.

- We have also included updated text for the "funding statement" and "role of funder" in the cover letter as requested. In addition, we have updated the formatting to be consistent with Plos One requirements.
